# TDM1 Regulation Determines the Number of Meiotic Divisions

**DOI:** 10.1371/journal.pgen.1005856

**Published:** 2016-02-12

**Authors:** Marta Cifuentes, Sylvie Jolivet, Laurence Cromer, Hirofumi Harashima, Petra Bulankova, Charlotte Renne, Wayne Crismani, Yuko Nomura, Hirofumi Nakagami, Keiko Sugimoto, Arp Schnittger, Karel Riha, Raphael Mercier

**Affiliations:** 1 Institut Jean-Pierre Bourgin, INRA, AgroParisTech, CNRS, Université Paris-Saclay, RD10, 78026 Versailles Cedex, France; 2 RIKEN Center for Sustainable Resource Science, Suehiro, Tsurumi, Yokohama, Japan; 3 Gregor Mendel Institute, Austrian Academy of Sciences, Vienna, Austria; 4 University of Hamburg, Biozentrum Klein Flottbek, Department of Developmental Biology, Hamburg, Germany; 5 Central European Institute of Technology (CEITEC), Masaryk University, Kamenice, Brno, Czech Republic; Fudan University, CHINA

## Abstract

Cell cycle control must be modified at meiosis to allow two divisions to follow a single round of DNA replication, resulting in ploidy reduction. The mechanisms that ensure meiosis termination at the end of the second and not at the end of first division are poorly understood. We show here that *Arabidopsis thaliana* TDM1, which has been previously shown to be essential for meiotic termination, interacts directly with the Anaphase-Promoting Complex. Further, mutations in *TDM1* in a conserved putative Cyclin-Dependant Kinase (CDK) phosphorylation site (T16-P17) dominantly provoked premature meiosis termination after the first division, and the production of diploid spores and gametes. The CDKA;1-CYCA1.2/TAM complex, which is required to prevent premature meiotic exit, phosphorylated TDM1 at T16 *in vitro*. Finally, while *CYCA1;2*/TAM was previously shown to be expressed only at meiosis I, TDM1 is present throughout meiosis. These data, together with epistasis analysis, lead us to propose that TDM1 is an APC/C component whose function is to ensure meiosis termination at the end of meiosis II, and whose activity is inhibited at meiosis I by CDKA;1-TAM-mediated phosphorylation to prevent premature meiotic exit. This provides a molecular mechanism for the differential decision of performing an additional round of division, or not, at the end of meiosis I and II, respectively.

## Author Summary

Meiosis is a fundamental process for sexually reproducing organisms that creates genetic diversity within populations. A key feature of meiosis is the reduction of the number of chromosomes, from two sets to one set, prior to fertilization. This reduction in chromosome number is due to two cell divisions following a single round of DNA replication. In this study, we analysed the mechanism which controls the number of cell divisions, ensuring that meiotic termination occurs after the second meiotic division, and not at the end of the first division. We used the model plant *Arabidopsis thaliana* to show that the gene *TDM1* has a central role in regulating meiotic cell divisions. The integrity of the gene affects whether one, two or three meiotic divisions will occur. We further explain the relationship between TDM1 and its regulator the cyclin TAM, and how they work together to produce reproductive cells with a reduced number of chromosomes. This tightly controlled mechanism ensures the transmission of the correct number of chromosomes from one generation to the next.

## Introduction

In the germ line of sexually reproducing organisms, a specialized cell division—meiosis—ensures ploidy reduction in the gametes. Accomplishment of meiotic chromosome segregation requires extensive modifications of cell cycle progression compared to mitosis: (i) a longer prophase where crossovers occur between homologues [1], and (ii) two rounds of chromosome segregation, preceded by a single round of DNA replication. Cyclin-dependent kinases (CDKs) promote progression through both meiosis and mitosis, and a central regulator of their activity is the anaphase-promoting complex/cyclosome (APC/C), a conserved multi-subunit E3 ubiquitin ligase that triggers the degradation of multiple substrates, including cyclins [2]. The modifications of the cell cycle machinery required for meiosis are not fully understood, but the general perception is that during prophase I, the activity of CDK-cyclin complexes increase slowly until peaking at the onset of the first division. This activity drops when cyclins are degraded by the APC/C to allow the segregation of homologous chromosomes at anaphase I. This decay is not complete, although it is sufficient to allow spindle disassembly, entry into a second meiotic division and the avoidance of intervening DNA replication. CDK-cyclin activity increases again at meiosis II, followed by a complete abolishment of this activity by the APC/C that allow sister chromatids to segregate to opposite poles and meiosis termination (reviewed in [2–4]). Thus, one critical aspect of the meiotic cell cycle is the meiosis I to meiosis II transition, where CDK activity has to decrease to trigger meiotic spindle disassembly, but be kept at a sufficiently high level to prevent DNA replication. Further, the mechanisms that ensure the entry into a second division must be turned off at the end of meiosis II to avoid the entry into a third division and ensure meiotic exit. The proteins and mechanisms that regulate these key meiotic transitions are very diverse among the studied eukaryotes (*Saccharomyces cerevisiae*, *Schizosaccharomyces pombe*, *Drosophila melanogaster*, *Mus musculus*, *Xenopus laevis* and *Arabidopsis thaliana*), even though they all directly modify the CDK-cyclin-APC/C module [5–13].

*A*. *thaliana* has at least five cell cycle CDKs (CDKA;1, CDKB;1, CDKB1;2, CDKB2;1 and CDKB2;2) and more than 50 cyclins, of which only a few have clear meiotic functions. CDKA;1 is a major cyclin-dependent kinase that drives meiotic progression in plants [14]. Though the core cyclins(s) that directly regulate meiotic progression remain to be identified, several cyclins have been shown to play a role at meiosis. The cyclin SDS is required for the formation of meiotic crossovers and acts together with CYCB3;1 in suppressing premature cell wall synthesis [15–17]. TAM, an A-type cyclin (CYCA1;2) is essential to prevent meiosis termination at the end of the first division [14,18,19]. In the *tam1* null mutant a single division occurs at meiosis, leading to the production of diploid gametes. The same defects are observed in *osd1*. OSD1 is an APC/C inhibitor and is the functional equivalent of Mes1 in fission yeast and Emi2/Erp1 in vertebrates [18,20–22]. Another component involved in meiotic progression is SMG7, a protein involved in nonsense-mediated RNA decay (NMD) in somatic tissues that is also essential for progression through anaphase II [23,24]. Finally, TDM1/MS5 (THREE DIVISION MUTANT1/MALE STERILE 5) is required for meiotic exit, its mutation leads to a failure to terminate meiosis after meiosis II and to an aberrant third division [25,26]. TDM1 is a protein of unknown function that shares limited structural similarities with the APC/C subunit CDC16/Cut9/APC6 [20]. Intriguingly, the expression of a non-destructible version of TAM, whose wild type version is only expressed during the first division [23], dominantly provokes the entry into a third meiotic division, mimicking the phenotype of the recessive loss-of-function *tdm1* mutant [20]. This suggested that TAM and TDM1 could be functionally related, but the nature of this relationship and the role of these two proteins were elusive. In this study we shed new light on the role and regulation of TDM1 during the meiotic cell cycle. We propose that TDM1 stimulates the APC/C to promote termination of meiosis, this activity of TDM1 being inhibited at meiosis I by CDKA;1-TAM phosphorylation to prevent premature termination of meiosis. These molecular data exemplify how CDK phosphorylation is important for the integrity of the meiotic program in plants.

## Results

### A genetic screen for mutants skipping the second meiotic division

To identify genes controlling meiotic progression, a genetic screen was designed based on the idea that mutations that prevent a second meiotic division—such as *osd1* and *tam*–would restore the fertility of mutants that have unbalanced chromosome segregation only at the second meiotic division [18,21]. This is the case for *spo11-1 rec8* double mutants, in which the first meiotic division resembles a mitosis (balanced segregation of sister chromatids to opposite poles) but the second division is unbalanced and leads to aneuploid gametes and hence very limited fertility [27]. Mutations in *OSD1* or *TAM*, that lead to meiotic exit before meiosis II, are indeed able to restore fertility of *spo11-1 rec8* [18,21]. Thus, a forward genetic screen based on the restoration of fertility of *spo11-1 rec8* was performed to identify genes whose mutations provoke a similar phenotype to *osd1* or *tam*. Despite their meiotic segregation defect and reduced fertility, *spo11-1 rec8* plants produced a sufficient amount of seeds for mutagenesis with ethylmethane sulfonate (EMS). The M1 generation, that is presumably heterozygous for any given EMS-induced mutation, was self-fertilized and harvested in bulks of ~5 plants to produce M2 families. M2 families were screened for increased fertility compared to *spo11-1 rec8* non-mutagenized controls.

**Fig 1:**
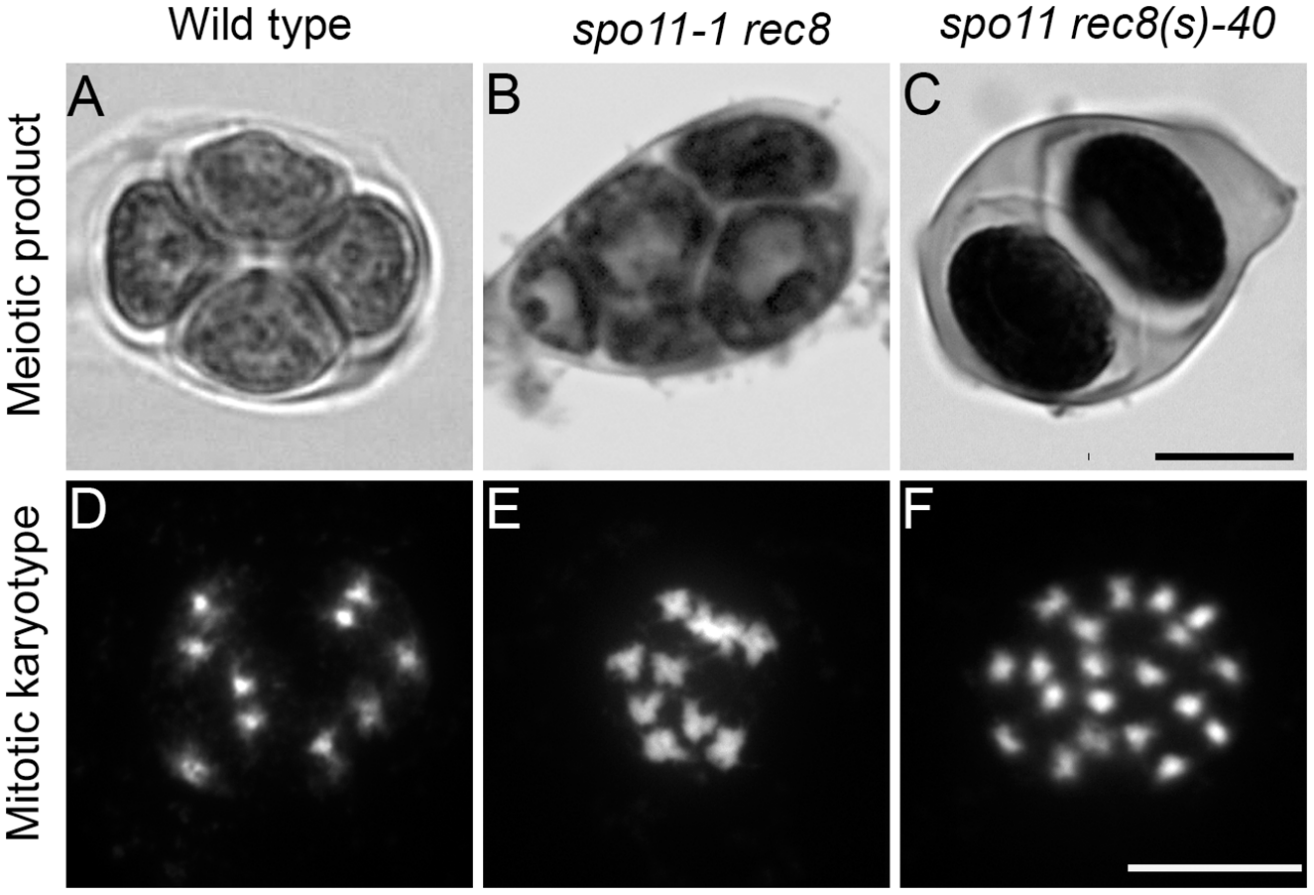
The *spo11-1rec8* (s)-40 mutant produces dyads and is tetraploid. (A to C). Male meiotic products stained by toluidine blue. (A) Wild type produces tetrads of spores. (B) *spo11-1 rec8* double mutant produces polyads (aberrant meiotic products with more than four spores). (C) *spo11-1 rec8 (s)-40* produces dyads of spores. (D to F) Mitotic karyotype. (D) Wild type is diploid, having ten chromosomes aligned on mitotic metaphase plates. (E) *spo11-1 rec8* is diploid. (F) The three *spo11-1 rec8* (s)-40 M1 plants were tetraploid, having 20 chromosomes aligned on mitotic metaphase plates. Scale bar = 10μM.

Three bulks segregated plants with increased fertility. Analysis of male meiotic products stained by toluidine blue showed that in all three cases, fertile plants produced dyads of spores, instead of tetrads that are observed in wild type, or polyads as observed in *spo11-1 rec8* (Fig 1) suggesting that the second meiotic division did not occur in those plants. Sequencing of candidate genes (*OSD1* and *TAM*) identified recessive mutations in *TAM* in two of the three families. The identified mutations were a splicing site in exon 7 (TAIR10 chr1:29082522 C>T) and a mutation in the 5’UTR region which introduced an upstream out of frame start codon (TAIR10 chr1:29084174 G>A). A complementation test showed that they were allelic, confirming that the mutations in *TAM* caused the dyad phenotype and the restoration of fertility. The third family (*spo11rec8(s)*-40) had no mutation in *OSD1* and *TAM* and is the focus of this study.

### A *TDM1* dominant mutation leads to a premature termination of meiosis

Chromosome spreads showed that the four fertile M2 plants from the same bulk identified in the screen (*spo11 rec8(s)*-40) were tetraploids (Fig 1). This suggested that the causal mutation was dominant and caused the production of male and female diploid gametes in the M1 plant. Whole genome sequencing of DNA pooled from two sister plants with ~100X coverage revealed the presence of 1,144 SNPs compared to wild type. However, only 15 SNPs appeared as homozygote. These few homozygote SNPs were located throughout the genome suggesting that they were present in the *spo11-1 rec8* line before mutagenesis, rather than resulting from fixation of *de novo* EMS-induced mutations. The fact that all other detected mutations were heterozygote further suggested that the causative mutation could be dominant. This dominant mutation would have been phenotypically expressed in the M1 plant, and due to combining this mutation with *spo11 rec8*, this would lead to the production of diploid clonal gametes. This phenocopies a *spo11-1 rec8 osd1* triple mutant (*MiMe*, [21]), hence maintaining heterozygosity of EMS induced mutations from the M1 plant in the tetraploid M2 plants.

**Fig 2:**
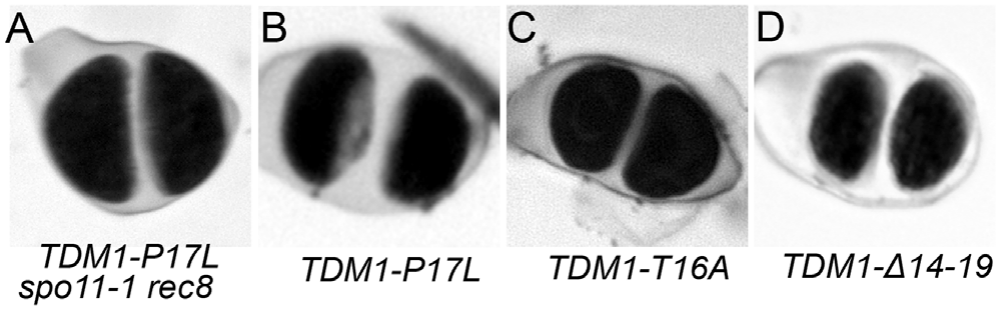
*TDM1-P17L*, *TDM116A* or *TDM1-Δ14_19* expression causes the production of dyads. (A) *spo11-1rec8* mutants transformed with *TDM1-P17L*. (B to C) Wild type plants transformed by (B) *TDM1-P17L*, (C) *TDM1-T16A or* (D) *TDM1-Δ14–19*. Dyads of spores were observed in all cases, compared to tetrads in wild type (Fig 1A). See Table 1 for quantification.

**Fig 3:**
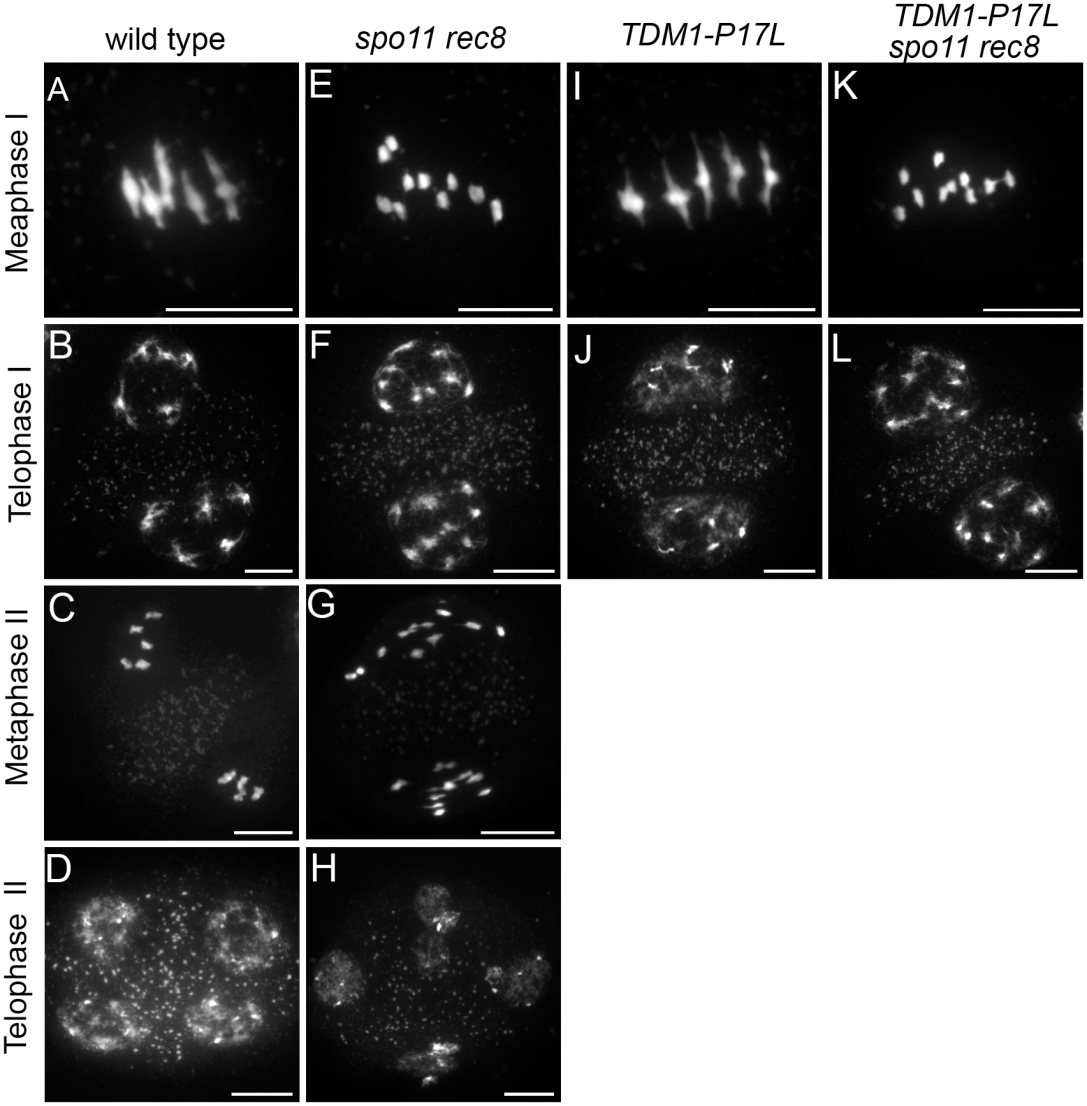
Meiotic chromosome spreads in wild type, *spo11-1rec8*, *TDM1-P17* and *spo11-1rec8 TDM1-P17* plants. (A to D) Meiosis in wild type. (A) Five bivalents align at metaphase I and (B) pairs of homologous chromosome are distributed into two nuclei at telophase I. (C) Five pairs of sister chromatids align on the two metaphase plates. (D) Four balanced nuclei are formed at telophase II. (E to H) Meiosis in *spo11-1rec8*. The first division resembles a mitotic division with (E) alignment of 10 pairs of chromatids on the metaphase plates and (F) segregation into two groups of 10 chromatids. (G) Single chromatids fail to align properly at metaphase II, resulting in (H) a variable number of unbalanced nuclei at telophase II. (I to J) Meiosis in wild type plants transformed with *TDM1-P17*. A single, meiosis I-like division is observed. (K to L) Meiosis in *spo11-1 rec8* plants transformed with *TDM1-P17*. A single, mitotic-like division is observed. Scale bar = 10μM.

Candidate causal mutations were then looked for among the heterozygote SNPs. Among these 1,129 mutations, 341 were predicted to affect a coding sequence (non-sense, missense or splicing site). A mutation in *TDM1* (TAIR10 Chr4:11185795 G>A) resulting in an amino acid change (*TDM1-P17L*), appeared as a good candidate as the potential causal dominant mutation as *TDM1* was previously shown to be essential for meiotic exit at the end of meiosis II. To test this hypothesis, a genomic clone containing *TDM1* (including promoter and terminator) that is able to complement *tdm1-3* mutant (Table 1) was mutated to recreate the mutation identified in the screen (*TDM1-P17L*). When introduced in *spo11-1 rec8* plants, the *TDM1-P17L* clone was able to restore fertility of primary transformants (Table 1. *spo11-1 rec8*: 0.1 seeds per fruit (n = 197), *spo11-1 rec8 TDM1-P17L*#15: 25 seeds per fruit (n = 15), *spo11-1 rec8 TDM1-P17L*#67: 48 seeds per fruit (n = 10), compared to 50 to 60 in wild type) and led to the production of dyads (Fig 2, Table 1). This shows that the mutation in *TDM1* is indeed the cause of restoration of fertility and dyad production in *spo11rec8(s)-40*. Analysis of meiotic chromosome spreads in *spo11-1 rec8 TDM1-P17L* primary transformants (Fig 3) showed a mitotic-like first division, with 10 univalents aligned at metaphase I and sister chromatids segregated at anaphase I (like in *spo11-1 rec8*), and an absence of the second division. Next, the ploidy level of *spo11-1 rec8 TDM1-P17L* primary transformants offspring was explored. Self-pollination of *spo11-1 rec8 TDM1-P17L* produced only tetraploids (4n) (Table 2). When *spo11-1 rec8 TDM1-P17L* was crossed with wild type as male or female, all of the progeny were triploid (Table 2). Altogether these results demonstrated that *spo11-1 rec8* mutants transformed by *TDM1-P17L* produce male and female mitosis-like derived spores, which result in functional diploid clonal gametes.

**Table 1:** Analysis of the effect of expression of mutated *TDM1* in wild type and a series of mutants. For each experiment, the total number of analysed primary transformants is indicated. A minimum of 50 cells were observed per primary transformant.

Construct	Transformed genotype	Number of independent transformants	Male meiotic products (number of transformants)	Interpretation
-	wild type	-	Tetrads	
-	*tdm1-3*	-	lobed monads	Attempt of third division
-	*osd1* or *tam-2*	-	Dyads	Omission of second division
-	*smg7*	-	-	Anaphase II arrest
-	*spo11-1rec8*	-	Polyads	Mitotic-like first division and unbalanced second division
*TDM1*	*tdm1-3*	11	(9) tetrads	Complementation
			(2) lobed monads	Lack of expression
*TDM1-P17L*	*spo11-1rec8*	3	(2) dyads	Mitotic-like first division and omission of second division
			(1) lobed monads	Likely co-suppression
	wild type	20	(14) dyads	Omission of second division
			(2) mix of dyads and tetrads	Omission of second division (partial)
			(4) lobed monads	Likely co-suppression
	*tdm1-3*	2	(2) dyads	Omission of second division
	*osd1*	13	(13) dyads	Omission of second division, No additive effect
	*tam-2*	14	(13) dyads	Omission of second division, No additive effect
			(1) lobbed monads	Likely co-suppression
	*smg7*	7	MII arrest	smg7 phenotype, *smg7* epistatic on *TDM1-P17*
*TDM1-T16A*	wild type	2	(2) dyads	Omission of second division
*TDM1-Δ14_19*	wild type	6	(3) dyads	Omission of second division
			(1) dyads and tetrads	Omission of second division (partial)
			(2) tetrads	Lack of expression
	*tdm1-3*	4	(4) dyads	Omission of second division
TDM1-Y14A	wild type	5	(5) tetrads	No effect of TDM1-Y14A
	*tdm1-3*	3	(2) tetrads	Complementation
			(1) lobed monads	Likely co-suppression
TDM1-P18A	Wild type	14	(14) tetrads	No effect of TDM1-P18A
TDM1-S62A	Wild type	7	(7) tetrads	No effect of TDM1-S62A
	*tdm1-3*	8	(5) tetrads	complementation
			(3) lobed monads	Lack of expression

**Table 2:** Ploidy analysis of *spo11-1 rec8 TDM1-P17 and TDM1-P17* offspring.

	Transformant	Selfed	Crossed as male with wild type	Crossed as female with wild type
*spo11-1 rec8 TDM1-P17L*	#15	100% 4n (n = 25)	100% 3n (n = 5)	100% 3n (n = 24)
	#67	100% 4n (n = 24)	100% 3n (n = 10)	100% 3n (n = 18)
*TDM1-P17L*	#1	100% 4n (n = 4)	nd	43% 3n, 57% 2n (n = 7)
	#2	60% 4n, 40% 3n (n = 30)	nd	5% 3n, 95% 2n (n = 18)
	#3	73% 4n, 27% 3n (n = 11)	nd	Nd
	#4	Nd	nd	27% 3n, 73% 2n (n = 15)
	#8	Nd	nd	22% 3n, 78% 2n (n = 18)

When introduced into wild type plants or *tdm1-3* mutants, the *TDM1-P17L* genomic clone provoked the production of dyads in primary transformants (Table 1, Fig 2). Male meiotic chromosome spreads showed that these plants had a wild type first division and an absence of the second meiotic division (Fig 3). Among progeny derived from self-pollinations of the primary transformants (which were diploid), only tetraploids and triploids were found (Table 2). When *TDM1-P17L* plants were fertilised with wild-type pollen grains, diploid and triploid plants were produced (Table 2). In summary, the *TDM1-P17L* mutation confers a similar meiotic defect as the recessive null *osd1* or *tam* mutations [18,21], leading to premature exit from meiosis before the second division and consequently to the production of diploid male and female gametes.

### TDM1 is regulated by T16 phosphorylation

*TDM1* belongs to a family of proteins conserved in plants. The *A*. *thaliana* genome contains five other genes having significant sequence similarity with *TDM1* (S1 and S2 Figs). The function of these TDM1-like genes is so far unknown. The closest homologue, TDM1-like1 (AT5G44330), originates from a duplication found in the *Brassicaceae* (S1 Fig). Two T-DNA mutants in which TDM1-like1 is disrupted (GABI_750C08 and GABI_173C10) were indistinguishable from wild type with respect to fertility and the formation of proper tetrads. Furthermore, the *tdm1 tdm1-like1* double mutants were indistinguishable from *tdm1* with meiocytes attempting a third division. Taken together, this suggests that *TDM-like1* is not essential for meiosis.

Sequences analyses showed that the *TDM1-P17L* mutation is in a highly conserved five amino-acid domain in the TDM1 and TDM1-like 1 protein subfamily (S1 Fig), and not present in the other TDM1-like proteins. Especially, P17 and the adjacent T16 are conserved in all members of the TDM1 and TDM1-like 1 subfamily (S1 Fig). This TP motif defines a minimum consensus CDK phosphorylation site with T16 being the phosphate receptor [28]. To test the role of this putative phosphorylation site, we created versions of *TDM1* in which we substituted T16 with a non phosphorylatable alanine (designated *TDM1-T16A*), or by deleting the entire conserved domain between amino acid 14 and 19 (*TDM1-Δ14_19*). When introduced into wild-type plants, both *TDM1-T16A* and *TDM1-Δ14_19* led to the production of meiotic dyads instead of tetrads in primary transformants, recapitulating the effect seen in *TDM1-P17L* (Table 1; Fig 2). Moreover, when introduced into *tdm1-3* mutants, *TDM1-Δ14_19* also provoked the production of dyads (Table 1). However, mutating tyrosine 14 (*TDM1-Y14A*) or proline 18 (*TDM1-P18A*), two less conserved amino acids (S1 Fig), did not affect meiosis in the wild type and was able to complement the *tdm1-3* mutation (Table 1), suggesting that tyrosine 14 and proline 18 tyrosine do not play crucial roles in the function of TDM1. In summary, expression of *TDM1*-*P17L*, -*T16A* and -*Δ14_19* mutations are equally able to dominantly confer premature meiosis exit. As TPs are potential phosphorylation sites, these results suggest that *TDM1* is regulated by phosphorylation to ensure the meiosis I to meiosis II transition.

**Fig 4:**
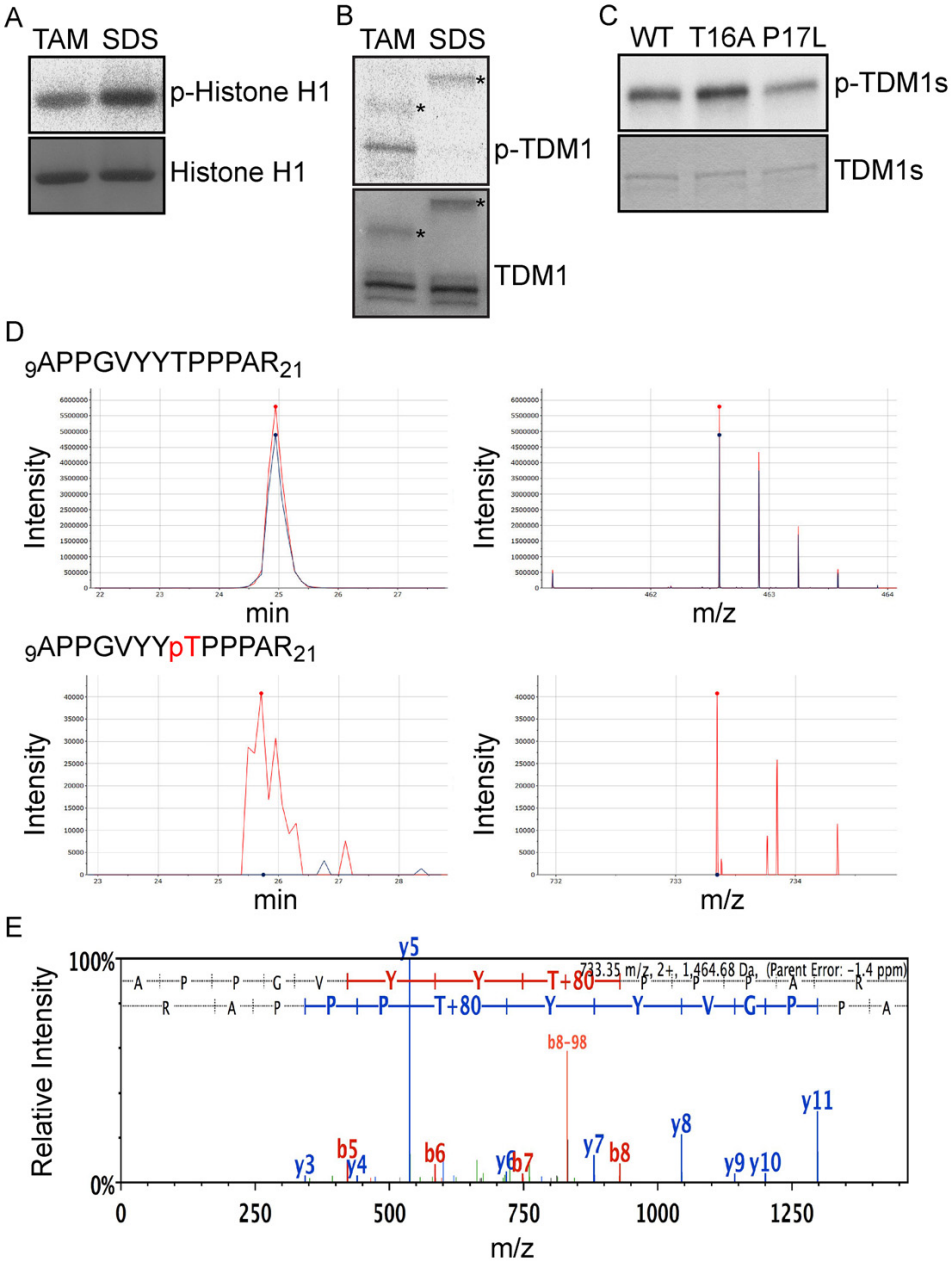
The CDKA;1-TAM complex phosphorylates TDM1 at threonine 16 *in vitro*. (A-C) Kinase essays. Proteins were subjected to a SDS-PAGE after the kinase reaction and stained with coomassie brilliant blue demonstrating equal loading of the substrate (bottom). (A) Histone H1 kinase assays. CDKA;1-TAM (TAM) and CDKA;1-SDS (SDS) complexes were subjected to kinase assays against histone H1. Phosphorylated histone H1 was detected by autoradiograph (top). Abbreviations: p-Histone H1 for radio-labeled histone H1 resulting from kinase assays with radio-labeled ATP. (B) TDM1 kinase assays. The same amount of the kinases used in (A) was subjected to kinase assays against GST-TDM-His6. Phosphorylated TDM1 was detected by autoradiograph (top). Abbreviations: p-TDM1 for radio-labeled TDM1 resulting from kinase assays with radio-labeled ATP. Asterisks indicate cyclin subunits. (C) TDM1 kinase assays. CDKA;1-TAM was subjected to kinase assays against wild-type and mutant TDMs. Phosphorylated TDM1 proteins were detected by autoradiograph (top). Abbreviations: p-TDMs for radio-labeled TDMs resulting from kinase assays with radio-labeled ATP. (D) TDM1 proteins treated with and without CDKA;1-TAM were subjected to mass spectrometry analysis. Chromatograms of selected ions representing two peptides (left) and mass ions of these peptides (right). The phosphopeptide 9-APPGVYYpTPPPAR-21 was detected only in TDM1 treated CDKA;1-TAM (red), but not in TAM without the kinases (blue) (bottom), whereas the corresponding non-phosphorylated peptide, 9-APPGVYYTPPPAR-21, was observed in both samples (top). pT (red) in the peptide sequence indicates the phosphorylated threonine. (E) MS/MS spectra of a phosphopeptide (9-APPGVYYpTPPPAR-21) from TDM1 treated with CDKA;1-TAM. The b and y ion series represent fragment ions containing the N- and C-termini of the peptide, respectively.

Previous work revealed a role for the cyclins TAM and SDS in meiosis and both were found to build active complexes with CDKA;1 [20,23]. *In vitro* kinase assays showed that TDM1 is phosphorylated by CDKA;1-TAM and not, or to very low levels, by CDKA;1-SDS, while both kinase complexes were active against a generic substrate (Fig 4A and 4B). Mass spectrometry of *in vitro* phosphorylated TDM1 identified peptides that contained a phosphorylated T16 residue corroborating that TDM1 is indeed a substrate of CDKA;1-TAM (Fig 4D and 4E). Conversely, we did not identify any peptides containing phosphorylated T16 when performing mass spectrometry analyses of TDM1-P17L treated with CDKA;1-TAM (S3 Fig). This, together with the meiotic defect conferred *in vivo* by *TDM1-T16A* and *TDM1-P17L*, suggests that the TDM1 function is regulated by CDKA;1-TAM through T16 phosphorylation.

In addition to T16, mass spectrometry revealed three other phosphorylated sites upon treatment with CDKA;1-TAM, S62, S253, and S320. This is consistent with the observation that TDM1-T16A and TDM1-P17L can be still phosphorylated *in vitro* by CDKA;1-TAM (Fig 4C). Peptides including phosphorylated version of these three amino acids were also identified when TDM1-P17L was treated with CDKA;1-TAM. However, the residues S253/P254 or S320/T321 are poorly conserved in the TDM1 proteins of flowering plants (S1 Fig). In addition, the expression of a non-phosphorylatable version of TDM1 at S62 (*TDM1-S62A*) in the null *tdm1* mutant restored meiosis (Table 1), demonstrating that the S62 residue does not play an essential role in regulating meiosis progression *in vivo*.

Our previous work showed that CDKA;1-TAM also phosphorylates OSD1 *in vitro* [20], suggesting that CDKA;1-TAM could regulate the function of OSD1 in addition to TDM1. OSD1 possesses seven potential CDK-dependent phosphorylation sites ([S/T]-P). The expression of *OSD1* mutated in all seven sites (S/T to A), complemented the null *osd1* mutant (S4 Fig), suggesting that the phosphorylation of OSD1 by CDKs does not play an essential role in meiotic progression.

### Epistasis analysis of *TDM1-P17L* with *osd1*, *tam*, *tamΔD* and *smg7*

**Fig 5:**
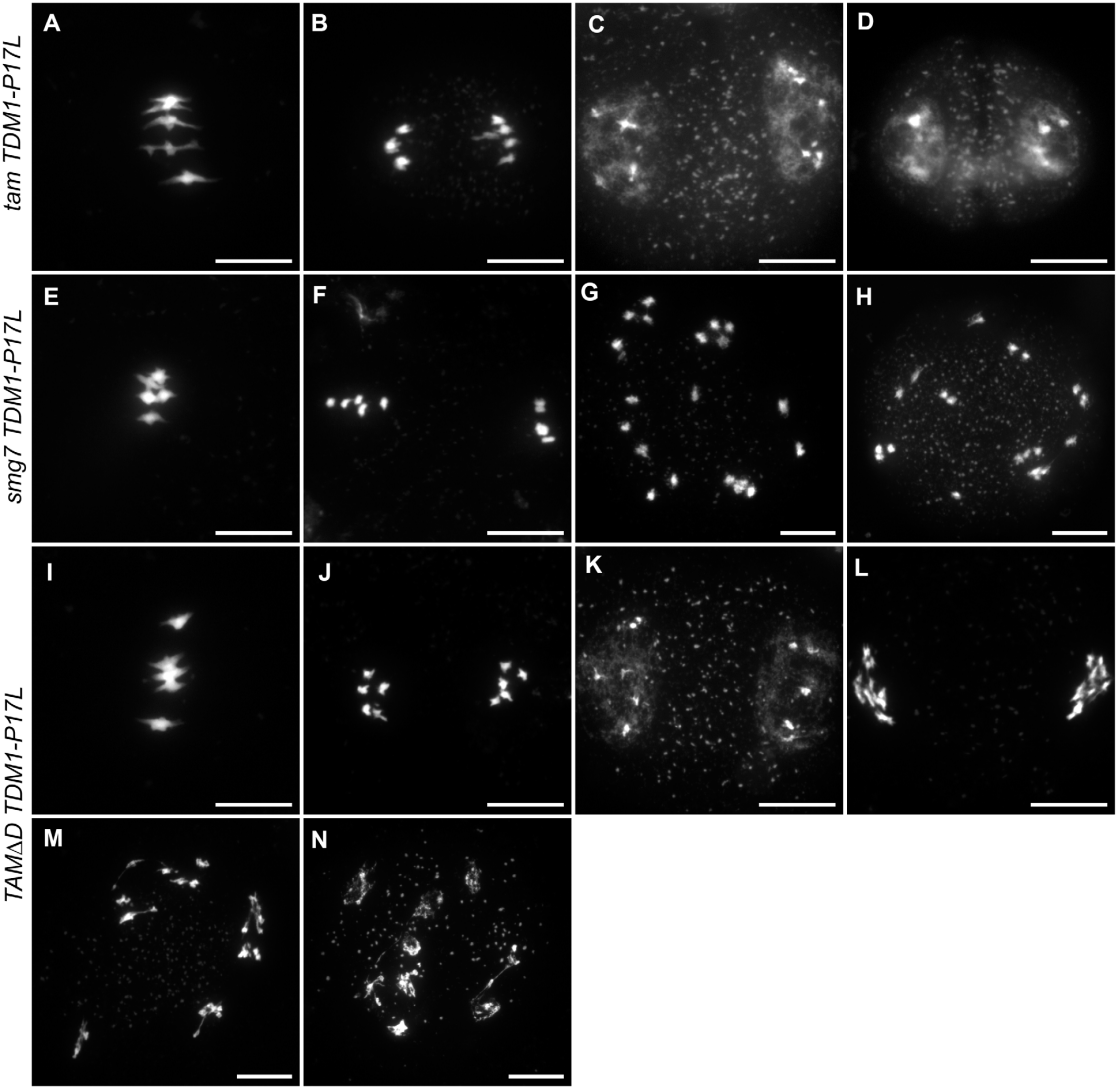
Meiotic chromosome spreads in *tam TDM1-P17L*, *smg7 TDM1-P17L and TAMΔD TDM1-P17L*. (A to D) *tam TDM1-P17L*. (A) Metaphase I. (B) Anaphase I. (C) Telophase I. (D) dyad. No second division was observed. (E to H) *smg7 TDM1-P17L*. (E) Metaphase I. (F) Metaphase II. (G-H) Arrest at anaphase II. (I to N) *TAMΔD TDM1-P17L*. (I) Metaphase I. (J) Anaphase I. (K) Telophase I. (L to N) Aberrant stages (see also Fig 6). No metaphase II, anaphase II or telophase II was observed. Scale bar = 10μM.

**Fig 6:**
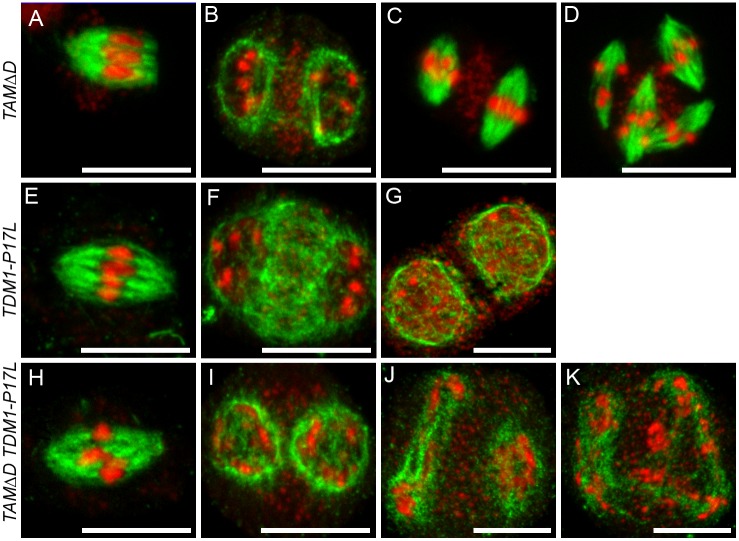
Tubulin immuno-localisation at meiosis *TAMΔD*, *TDM1-P17L* and *TAMΔD TDM1-P17L*. Tubulin appears in green and DNA in red. (A-D) *TAMΔD*. (A) Metaphase I. (B) Telophase I. (C) Metaphase II. (D) Metaphase III with the formation of four spindles. (E-F) *TDM1-P17L*. (E) Metaphase I. (F) Telophase I (G) Dyad of spores. No second division is observed. (H-K) *TAMΔD TDM1-P17L*. (H) Metaphase I. (I) Telophase I. (J) Aberrant stage with apparent uncoupling of spindle formation and chromosome condensation. (K) Aberrant stage with chromosomes scattered in the cell. No figures of meiosis II or meiosis III were observed. Scale bar = 10μM.

To further investigate the role of *TDM1* and its interactions with the other components of the meiotic cell cycle we performed epistasis analyses (Table 1, Figs 5 and 6, Table 3). When the *TDM1-P17L* construct was introduced into *tam-2* or *osd1-3* mutants, the transformants produced dyads at the end of a regular meiosis I (Table 1 and Fig 5A–5D), as occurs in each single mutant. Thus the *TDM1-P17L* mutation does not modify the *osd1* or *tam* null mutant defects, and *vice versa*.

**Table 3:** Summary of *TAM-TDM1-OSD1-SMG7* epistasis interactions.

TAM	TDM1	OSD1	SMG7	Number of divisions	Comments	references
wt	wt	wt	wt	2	-	-
**null**	wt	wt	wt	1	omission of second division	[18]
wt	**null**	wt	wt	3	attempted third division	[25,26]
wt	wt	**null**	wt	1	omission of second division	[21]
wt	wt	wt	**null**	2	arrest at anaphase II	[24]
**TAMΔD**	wt	wt	wt	3	attempt of third division	[20]
wt	**TDM1-P17L**	wt	wt	1	omission of second division	This study
**null**	**null**	wt	wt	3	attempted third division	[23]
**null**	Wt	**null**	wt	0	no division	[18,20]
wt	**null**	wt	**null**	3	attempted third division	[20]
wt	**null**	**null**	wt	1	omission of second division	[20]
wt	Wt	**null**	**null**	1	omission of second division	[20]
**null**	Wt	wt	**null**	2	arrest at anaphase II	[23]
**TAMΔD**	**null**	wt	wt	3	attempted third division	[20]
**TAMΔD**	Wt	**null**	wt	1	arrest at telophase I	[20]
**null**	**TDM1-P17L**	wt	wt	1	omission of second division	This study
wt	**TDM1-P17L**	**null**	wt	1	omission of second division	This study
wt	**TDM1-P17L**	wt	**null**	2	arrest at anaphase II	This study
**null**	**null**	**null**	wt	1	arrest at telophase I	[20]
**TAMΔD**	**TDM1-P17L**	wt	wt	1	Aberrant meiosis II	This study

SMG7 is essential for progression through meiosis II, the *smg7-1* mutant being arrested at anaphase II [23,24]. When the *TDM1-P17L* construct was introduced into *smg7-1*, the transformants had the *smg7-1* phenotype (Table 1 and Fig 5E–5H): meiosis was indistinguishable from wild type until metaphase II (Fig 5E and 5F) but an anaphase II arrest was observed with an incapacity to distribute the chromatids (Fig 5G and 5H), resulting in complete sterility. This shows that the *smg7-1* mutation is epistatic on the *TDM1-P17L* mutation. Finally, we co-transformed wild type plants with *TDM1-P17L* and a non-destructible version of *TAM* (*TAMΔD* [20]), and selected primary transformants carrying either both or one of the transgenes (Figs 5 and 6). Plants transformed only with *TAMΔD* (n = 6/6) were sterile, and meiocytes went through both meiosis I and meiosis II, before attempting a third meiotic division characterized by the formation of four spindles (Fig 6A–6D), as previously described [20]. Plants transformed with only *TDM1-P17L* (n = 8/9) were fertile and produced dyads following the first division (Fig 6E–6G), as described above. Plants transformed with both *TDM1-P17L* and *TAMΔD*, were sterile and showed a meiotic defect that differed from both single transformants: In these plants (n = 8/8), meiocytes went through normal meiosis I until telophase I (Figs 5I–5K and 6H–6I). Neither regular meiosis II nor meiosis III were observed, but aberrant meiosis II-like figures (Figs 5L–5N and 6J and 6K) with uncondensed and stretched chromosomes, ending with chromosomes being scattered throughout the cell and complete sterility. This shows that expressing *TDM1-P17L* in *TAMΔD* prevents the occurrence of a second and third meiotic division and, conversely, that the expression of *TAMΔD* in *TDM1-P17L* prevents meiotic termination after meiosis I, forcing cells entering into an aberrant meiosis II.

### TDM1 is present throughout meiosis

**Fig 7:**
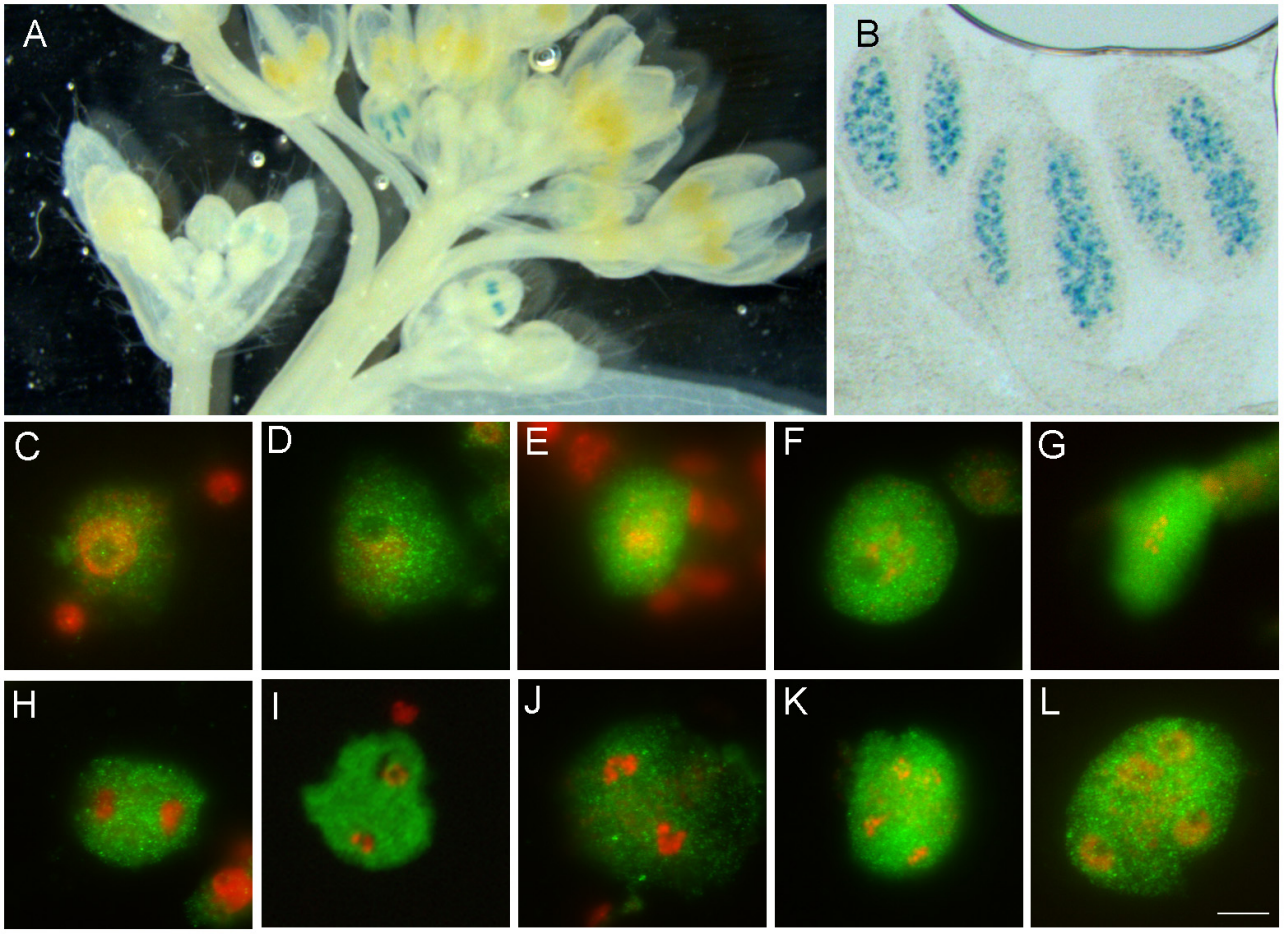
TDM1 localization. (A,B). GUS histochemical assays on plants transformed by *TDM1*:*GUS*. (A) The signal (blue) is detected in anthers of young buds, corresponding to the meiotic stage. (B) A close up of a group of anthers showing that the signal is detected in meiocytes. (C-L). Immunolocalization of TDM1::Myc (green). The DNA appears in red. TDM1::Myc is detected from mid-prophase to the tetrad stage. See S5 Fig for specificity of the signal.

While TAM is specifically expressed in meiosis I, TDM1 was identified as a protein required for meiotic exit [23]. Nevertheless, our genetic and biochemical data indicate that TAM and TDM1 functionally interact and that TDM1 may also be expressed in meiosis I. To examine TDM1 expression, we generated transgenic plants carrying the entire *TDM1* gene fused to a β-glucuronidase (GUS) reporter at the C terminus. GUS histochemical assays revealed that *TDM1*::*GUS* was specifically expressed in anthers of flower buds whose size correspond to the meiotic stage (Fig 7A and 7B). Further, a *TDM1*::*Myc* genic fusion was constructed and shown to be able to complement the *tdm1* mutation. Myc Immuno-localization on male meiocytes showed that TDM1::Myc was present throughout both meiotic divisions, from mid-prophase to the tetrad stage (Figs 7C–7L and S6). This data indicates that expression of TAM and TDM1 partially overlaps in meiosis I[23].

### *TDM1* interacts with APC/C subunits

**Fig 8:**
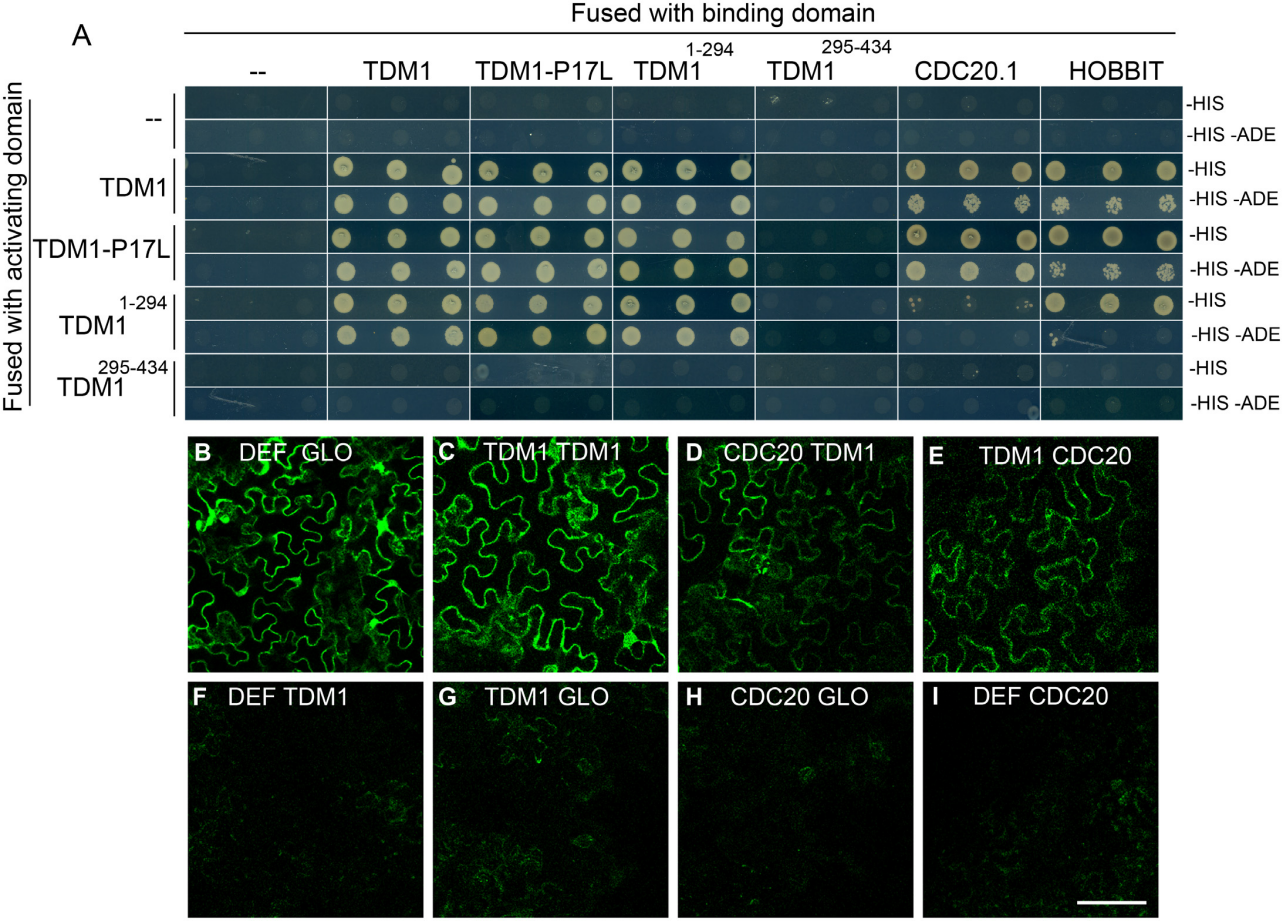
TDM1 interacts with itself, CDC27a and CDC20.1. (A) Yeast two-hybrid. Interaction between the bait fused with the GAL4 DNA binding domain (pDEST32) and the pray fused with the GAL4 activation domain (pDEST22) is revealed by growth on media depleted of histidine (-his, weak interaction) or media depleted of both histidine and adenine (-His—Ade, strong interaction). Three replicates are shown for each test. (B to I) Bimolecular fluorescent complementation (BiFC). Interaction between two proteins is revealed by reconstituting the fluorescence of split YFP, in pavement cells that are shaped like the pieces of a jigsaw puzzle. (A) Positive control: YFP^N^::DEF and YFP^C^::GLO, two interacting components of the *Anthirrinum majus* MADS box transcription factors DEFICIENS and GLOBOSA. (B) YFP^N^::TDM1 and YFP^C^::TDM1. (C) YFP^N^::TDM1 and YFP^C^::TDM1. (D) YFP^N^::TDM1 and YFP^C^::CDC20.1. (E) YFP^N^::DEF and YFP^C^::CDC20.1. (F) YFP^N^::DEF and YFP^C^::TDM1. (G) YFP^N^::TDM1 and YFP^C^::GLO. (H) YFP^N^::CDC20.1 and YFP^C^::GLO. (I) YFP^N^::DEF and YFP^C^::CDC20.1. Scale bar = 100μm.

TDM1 contains a four-TPR (tetratricopeptide repeat, 61–224) domain with structural similarity to the TPR domains of the APC/C subunits APC6, APC3, APC and APC8 [20], suggesting that TDM1 could interact with or be a subunit of the APC/C. To test this hypothesis we used Y2H experiments to assess interaction of TDM1 with different APC/C subunits (Fig 8A). In this assay, TDM1 interacted with itself and this interaction was mediated by its N terminal (1–294), which contains the TPR domains. TPR domains are known to mediate protein-protein interactions, and notably self-dimerization in the case of the TPR-containing APC/C subunits [29,30]. In addition, TDM1 interacted with the APC/C core component CDC27b (HOBBIT/APC3b) and the APC/C activator CDC20.1. No interaction was detected between TDM1 and the other core subunits tested (CDC27a, APC5, APC6, APC7, APC8, APC10, APC11), nor other APC/C activators (CDC20.3, CCS52A1, CCS52A2, CCS52B or SAMBA), nor OSD1 nor PANS1 [31]. TDM1 interactions with itself and with CDC20.1 were confirmed by bimolecular fluorescent complementation (BiFC) experiments (Fig 8B–8I). Finally, the TDM1-P17L version was able to interact in yeast two-hybrid with itself, CDC20.1, HOBBIT and TDM1, like wild type TDM1 (Fig 8A), suggesting that TDM1 Pro17 is not essential neither for TDM1 dimerization nor for the interaction of TDM1 with the APC/C.

## Discussion

Previous data showed that TAM and TDM1 are both key players of the transitions between meiotic divisions. TDM1 promotes meiotic termination after the second meiotic division, its absence provoking the entry into an aberrant third meiotic division [23]. Conversely, TAM promotes the transition from the first to the second meiotic division, as its absence provokes a premature termination of meiosis at the end of meiosis I [18]. The expression of a non-destructible version of TAM dominantly provokes the entry into a third meiotic division, similar to the *tdm1* knockout mutant [20]. This suggested that TAM and TDM1 could be functionally related, but their function and mechanisms of action remained to be unravelled.

### TDM1 is a putative meiotic APC/C component

**Fig 9:**
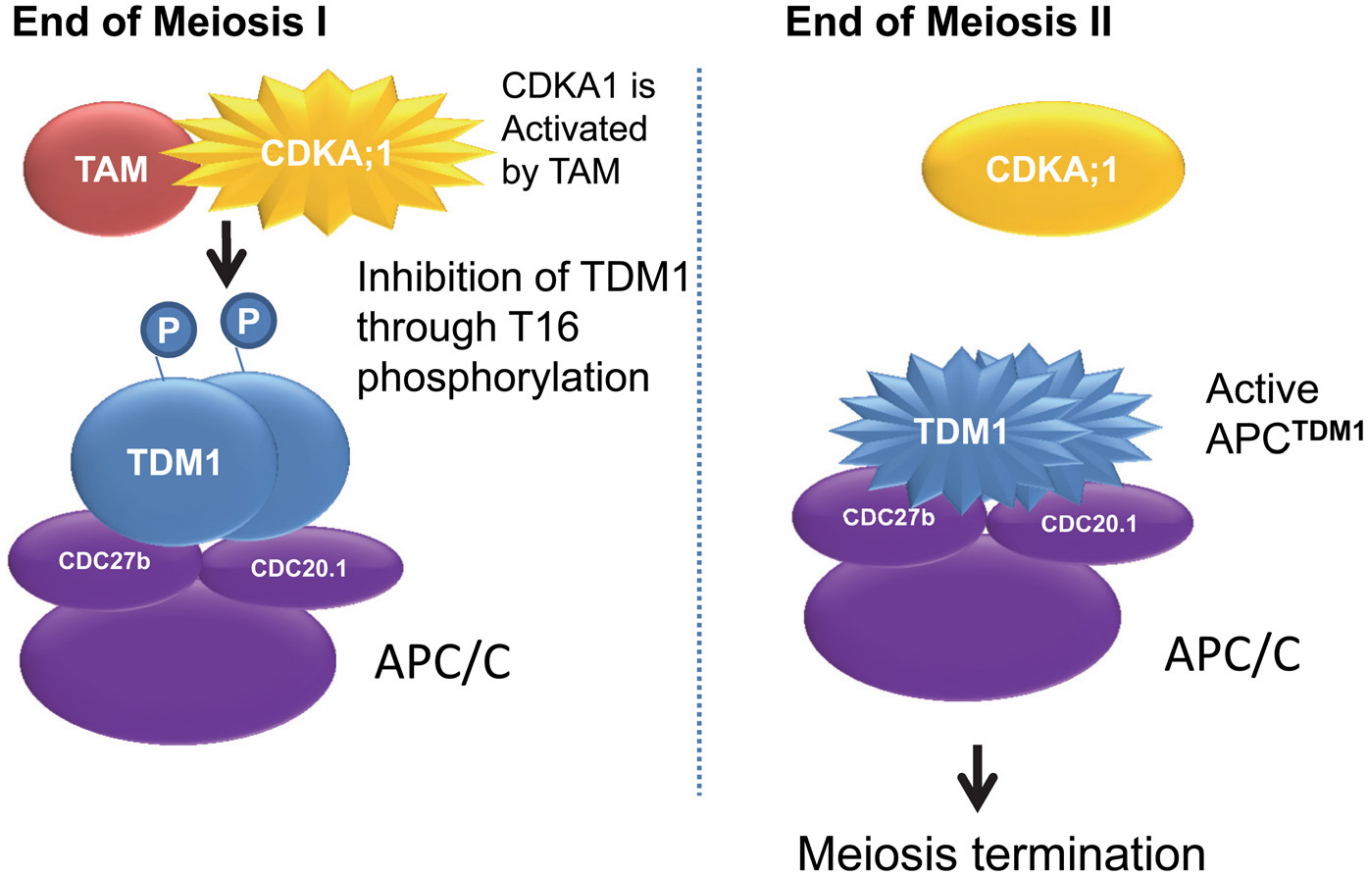
Model of the regulation of meiosis progression by TDM1 and TAM.

Control of APC/C activity is fundamental to regulate cell cycle progression and termination. The APC/C ubiquitinates cyclins and other targets to trigger their degradation by the proteasome promoting anaphase I and cell cycle exit. Here we propose that TDM1 is an APC/C meiotic component. TDM1 shares structural similarities with the TPR-containing APC/C subunits [20]. TDM1 interacts with itself, suggesting that it acts as a homodimer like the TPR-containing APC/C subunits. Interestingly, a *tdm1* mutation (*ms5-2*) that truncates the protein by 112 amino acids at the C-terminus, leaving intact the TDM1-TDM1 interaction domain, dominantly provokes the loss of function phenotype [26], which is compatible with TDM1 acting as a homodimer because the non-functional version of TDM1 would sequester functional versions of TDM1 into a non-functional complex. Further, yeast two-hybrids and BiFC assays showed that TDM1 interacted directly with the APC/C activator CDC20.1 and with the TPR-containing APC/C core component CDC27b (HOBBIT). Together with the absence of meiotic exit provoked by the *tdm1* loss-of-function, this suggests that TDM1 could promote meiotic termination by activating the APC/C and/or by modifying its specificity (Fig 9). This would trigger the elimination of the remaining cyclins from the cell and promote the exit from the meiotic program. As TDM1 is present throughout meiosis, its activity must be negatively regulated to prevent premature meiotic termination before the end of meiosis II.

### A TAM-TDM1 bipartite functional module controls MI-MII and MII-exit transitions of meiosis

Here we showed that mutating the TDM1 Proline 17 (*TDM1-P17L*) or its adjacent Threonine 16 (*TDM1-T16A*) dominantly provokes an exit from meiosis after the first division, mimicking the phenotype of the recessive *tam* loss-of-function mutant. Further, TAM in combination with the cyclin-dependent kinase CDKA;1 is able to phosphorylate TDM1 at T16 *in vitro*. In addition, the TDM1 protein is present throughout meiosis, while TAM is present only at meiosis I. We also showed that TDM1 interacts directly with the APC/C. Altogether, this strongly suggests a model in which CDKA;1-TAM inhibits TDM1 at meiosis I through T16 phosphorylation, preventing premature meiotic exit (Fig 9 left panel). At the second division, the absence of TAM would reactivate TDM1, and this would promote meiotic exit at the end of meiosis II via APC/C activation (Fig 9 right panel). This model accurately predicts the premature meiosis exit observed in *tam*, *TDM1-T16A* or *TDM1-P17L*, and the failure to exit from meiosis after the second division of *tdm1* or *TAMΔD*, and is further supported by epistasis data (Table 3): as predicted, (i) the null *tdm1* mutation in *tam* suppressed premature exit from meiosis and provoked the entry into a third meiotic division; (ii) Expressing TDM1-P17L in *tam* did not modify the premature meiotic exit seen in both single mutants; (iii) Expressing TAMΔD in *tdm1* did not modify the three-division meiotic defect observed in both single mutants.

This simple two-component model would predict that expressing both TAMΔD and TDM1-P17L should lead to a single meiotic division, because the expression of a non-destructible version of TAM would not affect the precocious meiotic exit provoked by TDM1-P17L, which is resistant to TAM-mediated regulation. However, in plants expressing both TDM1-P17L and TAMΔD, meiosis did not end after meiosis I like in TDM1-P17L, but arrested at an aberrant stage. This suggests that TAM has target(s) other than TDM1-P17 (see below also).

Given the diversity in the regulation of APC/C across kingdoms [2], the mechanisms of APC/C regulation for meiosis termination seem far from universal. TDM1 seems to be the functional equivalent to Mfr1/Fzr1 in fission yeast. Termination of meiotic divisions is dependent on activation of the APC/C by Mfr1/Fzr1. It has been shown that Cuf2 up-regulates Mfr1/Fzr1 in meiosis II to activate APC/C activity, degrade cyclins and lead to exit from meiosis. Both *cuf2*, *fzr1* and *cuf2 fzr1* mutants undergo an aberrant third division where chromatids try to divide again [32]. As TDM1 is also present in meiosis I, it must be kept latent to avoid premature meiosis termination. We propose that only the relief of its inhibition at the second meiotic division promotes APC/C activity to completely shut off CDK activity. Differences in the mode of regulation of these genes in fission yeast and plants would have converged to deliver the same outcome of APC/C regulation and meiosis termination (Fzr1 is regulated at the transcriptional level by the transcription factor Cuf2, while TDM1 is regulated post-translationally by CYCA1;2/TAM).

### The functional relationship between the TAM-TDM1 module and other regulators of meiotic progression

OSD1, a functional homolog of the fission yeast Mes1 [7], promotes entry into the second meiotic division through APC/C inhibition [20,22]. In addition, OSD1 and TAM act in a synergistic manner to promote the transition from prophase to the first meiotic division, since the double mutant *tam osd1* is not able to enter the first meiotic division [18]. Here we showed that, in contrast to *tam*, expression of TDM1-P17L in *osd1*, does not affect the entry into meiosis I as meiocytes exit meiosis after the first division, as in *osd1* mutants. This suggests that TAM promotes entry into meiosis I independently of both TDM1-P17 and OSD1. TAM can phosphorylate OSD1 *in vitro* [20]. However, a version of *OSD1* mutated in its seven potential CDK-dependent phosphorylation sites ([S/T]-P) is still functional, raising doubts about the relevance of this phosphorylation *in vivo*. Further work is required to identify the additional target(s), beyond TDM1-P17L, of TAM.

SMG7 is essential for the progression of meiosis II, as *smg7-1* arrests at anaphase II. SMG7 has been proposed to control meiosis II exit through TDM1, as the *tdm1* mutation suppresses the *smg7* anaphase II arrest [20,23]. Neither *tam* [23] nor the TDM1-P17L mutations [this study] alleviate the anaphase II arrest in *smg7* mutant, which is consistent with our model (*tam* or *TDM1-P17L* is predicted to not affect meiosis II progression). By contrast, these double mutants show that *smg7* abolished the premature meiotic exit in *tam* and in *TDM1-P17L* plants. This suggests that *smg7* promotes meiotic progression by regulating TDM1 in parallel to TAM. Although SMG7 has been described as a nonsense-mediated RNA decay factor, previous work has shown that its role at meiosis is not mediated through this activity [33]. More work is required to understand the meiotic role of this multifaceted protein.

The regulation of TDM1 by CDKA;1-TAM-mediated phosphorylation determines the differential fate of first or second division meiocytes, ensuring the entry into the second meiotic division and meiotic exit. This sheds new light on the importance of CDK-mediated phosphorylation in meiotic cell cycle regulation. In addition to providing new insight into the regulation of meiotic progression, the identification of a dominant mutation that leads to premature meiotic exit, and that leads to the production of clonal gametes when combined with *spo11 rec8*, could facilitate the development of synthetic clonal reproduction through seeds (apomixis) [34].

## Materials and Methods

### Genetic material

The mutant alleles used in this study were *spo11-1-3*, *rec8-2*, *osd1-3*, *tam-2*, *tdm1-3*, *smg7-1* and were genotyped as in [20,21,24]. They are all in the same genetic background (Col-0). The TDM1-like1 alleles (GABI_750C08 and GABI_173C10) were obtained from the NASC. Plants were identified by PCR using primers as follow: GABI_750C08U (5’-AGGTTTCTTGACTCCACCACC-3’), GABI_750C08L (5’-CCGCTACTGTTGCTTGTTCTC-3’) and Lb (5’- CCCATTTGGACGTGAATGTAGACAC-3’).

### EMS mutagenesis and mutation identification

EMS mutagenesis was performed as previously described [35]. The M1 plants that are presumably heterozygous and chimeric for EMS mutations were self-fertilized and harvested in bulks of ~5 to produce M2 families. About 2000 M2 families (400 bulks) were screened for increased fertility compared to *spo11-1 rec8* non-mutagenized control. Whole genome sequencing was done by Illumina Highseq 2000 100pb paired ends (The Genome Analysis Centre, Norwich). A list of SNPs was generated compared to the reference genome of *A*. *thaliana* TAIR10 (cultivar Columbia) using the mutdetect pipeline [36].

### Cytology and ploidy analysis

Male meiotic products observation, chromosomes spreads, and ploidy measurement were carried out using the techniques described in d'Erfurth et al [37].

### Directed mutagenesis constructs and plant transformation

A TDM1 genomic fragment was amplified by PCR using TDM1 U (5′-GACATCGGCACTTGCTTAGAG 3′), TDM1 L (5′-GCGATATAGCTCCCACTGGTT-3′). The amplification covered 986 nucleotides before the ATG and 537 nucleotides after the stop codon. The PCR product was cloned, by Gateway (Invitrogen), into the pDONR207 vector (Invitrogen), to create pENTR-TDM1, on which directed mutagenesis was performed using the Stratagene Quickchange Site-Directed Mutagenesis Kit. The mutagenic primers used to generate mutated version of TDM1 were: *TDM1-P17L*: (5′GAGTTTACTATACTC**T**GCCGCCGGCGAGAAC-3′); *T16A*: (5′CTCCACCTGGAGTTTACTAT**G**C**C**CCGCCGCCGGCGAGA -3′); *TDM1-Y14A*: (5’-CCACCTGGAGTT**GCG**TATACTCCGCCGCGGCG-3′); and *TDM1-Δ14–19* (5’-CCACCTGGAGTTGCGAGAACAAGTGATCATGTGGC-3’); and their respective reverse complementary primers. The mutagenic primers used to generate mutated version of OSD were OSD1_T198A_U: GGAAGAAGCTGGCTTCATCGCACCCGAGAAGAAGC; OSD1_ T105A_U: GTTGCCTTCTTGGTATCCAAGAGCACCTCTACGCG; OSD1_T224A_U: GGCGGAGATCCAGAAGTTGAAGAGCGCTCCTCAAGCTA; OSD1_S40A_U: CACGGCTTAGTTTGATTGAAGCTCCGGTGAATCCAG; OSD1_S146A_U: GTTGGTGTTCTTGAAGCTCCAGTACCACTGTCAGG; OSD1_T160A_U: AAATGCTCGATGGTCGCTCCTGGACCATCTGTGGG; OSD1_T73A_U: TGGCAGAGGTGGTCACGCTCCATTTAGATTGCCAC; and their respective reverse complementary primers. To generate binary vectors for plant transformation, an LR reaction was performed with the binary vector for the Gateway system, pGWB1. The resulting binary vectors, pTDM1, pTDM1-P17L, pT16A, pTDM1-Y14A and pTDM1-Δ14–19 were transformed using the Agrobacterium-mediated floral dip method on wild type plants and plant populations segregating for *spo11-1 rec8*, *osd1-3*, *tam-2* or *tdm1-3* mutation. Transformed plants were selected on agar plates containing 20 mg/L hygromycin and relevant homozygous mutants were identified among primary transformants through genotyping. The *TAMΔD* construct [20] was cloned into the binary vector pMDC123 (www.arabidopsis.org) that confers BASTA resistance (BAR gene) and introduced in Agrobacterium. Double transformation of Col-0 wild type by *TAMΔD* and *TDM1-P17L* was performed by mixing the two agrobacterium cultures before floral dipping. Transformants were first selected *in vitro* on 20 mg/L hygromycin and then genotyped by PCR for the *BAR* gene to identify double transformants. Among 174 hygromycin resistant primary transformants, 85 were also transformed by the TAM*ΔD/BAR* transgene.

### Yeast two-hybrid assay

*TDM1* cDNA was amplified by PCR using 5’-ATGTGTCCCTGCGTAGAGCGT-3’ and 5’-CTACATCTCTGCGGTTTTAAGCTC-3’. *CDC20*.*1* cDNA was amplified by PCR using 5’-ATGGATGCAGGTATGAACAAC-3’ and 5’-TCAACGAATACGATTCACG-3’ *TDM1*^1-294^ cDNA was amplified by PCR using 5’- ATGTGTCCCTGCGTAGAGCGT -3’ and 5’- TATTTCTGCTAACATTTCG -3’. *TDM1*^295-434^ cDNA was amplified by PCR using 5’-CGAAATGTTAGCAGAAATAGA-3’ and 5’-CTACATCTCTGCGGTTTTAAGCTC-3’. PCR product was cloned by Gateway (Invitrogen) into the pDONR221 vector (Invitrogen) to create pENTR. The other APC/C clones were described in [38].The mutagenic primers used to generate mutated version of *TDM1-P17L* were the same as described above. LR reactions were done into the pDEST32 (bait) and pDEST22 (prey) vector (Invitrogen). Y2H interaction was performed by mating, as described previously [38] into the yeast strain AH109 and Y187 (Clontech).

### Bimolecular fluorescence complementation (BiFC)

Protein interactions were tested *in planta* using BiFC assays [39] in leaf epidermal cells of *N*. *benthamiana* [40]. N-terminal fusions, using the pENTR clones described above for Y2H, with two YFP complementary regions (YFP^N^ + YFP^C^) were co-infiltrated in *N*. *benthamiana* leaves and scored after 3 or 4 days for fluorescence as described in [41]. YFP^N^::DEF and YFP^C^::GLO [42] were used as positive controls for interaction. Each experiment was replicated at least three times. Observations were made using a Leica SP5 II AOBS Tandem HyD confocal laser-scanning microscope. Optical sections were collected with a Leica HCX PL APO CS 20.0x0.70 IMM UV water objective upon illumination of the sample with a 514-nm argon laser line with an emission band of 525–570 nm for the YFP. The specificity of the YFP signal was systematically checked by determining the fluorescence emission spectrum between 525 and 600 nm with a 10-nm window and under an excitation at 514 nm. Images were processed using Leica LASAF and Adobe Photoshop software.

### Kinase assay and MS analysis

Target genes were subcloned into an entry vector and recombined into the destination vector pGGWA [43] by using LR Clonase II (Life Technologies). Error-free destination clones were confirmed by sequence analyses. *E*. *coli* BL21-AI cells (Life Technologies) were transformed with the resulting destination clone and grown in LB medium containing 100 μg/ml ampicillin until OD_600_ = 0.6 at 37°C. The culture was transferred to 18°C and grown for 30 min. The production of the fusion protein was induced by adding 0.3 mM isopropyl-β-d-thiogalactopyranoside (IPTG) and 0.2% arabinose overnight at 18°C. Cells were harvested by centrifugation and re-suspended in Ni-NTA binding buffer (50 mM NaH_2_PO_4_, 100 mM NaCl, 10%(v/v) glycerol, 25 mM imidazole, pH 8.0) containing protease inhibitors (cOmplete ETDA-free; Roche), and lysed by sonication (Digital Sonifier 450D, BRANSON). After addition of Triton X-100 to 0.2%(w/v), the cell slurry was incubated at 4°C for 20 min then clarified by centrifugation. The supernatant was passed through a column packed with Ni-NTA resin (Qiagen), which was washed sequentially with Ni-NTA binding buffer, and eluted with Ni-NTA elution buffer (Ni-NTA binding buffer containing 200 mM imidazole). The eluate was applied onto a column packed with glutathione agarose (Sigma), which had been equilibrated by Ni-NTA buffer. The column was washed sequentially with Ni-NTA binding buffer followed by kinase buffer (50 mM Tris-HCl, pH 7.5, 10 mM MgCl_2_, 1 mM EGTA), then the fusion protein was eluted with kinase buffer containing 10 mM glutathione. After CDK complexes were expressed and purified by using a system as described [44], ATP was added to 2 mM, and the complexes were incubated for 1 h at 30°C. The reaction was then further purified with a column packed with Strep-Tactin sepharose resins (IBA), which had been equilibrated with kinase buffer. CDK complexes were eluted with kinase buffer containing 2.5 mM desthiobiotin. The aliquoted complexes were frozen in the liquid nitrogen and stored at -80°C.

The kinase assays were carried out with CDKA;1-TAM or CDKA;1-SDS as a kinase, 2 μg of histone H1^0^ (NEB) or purified GST-TDM1(WT, T16A, and P17L)-His6 as a substrate, 92.5 kBq of [γ-^32^P]ATP (PerkinElmer) per reaction in kinase buffer with a final volume of 20 μl. After incubation for 30 min at 30°C, the reactions were stopped by adding Laemmli sample buffer (Bio-rad) and boiled. Samples were separated on 12% (for Histone) or 7.5% (for TDM1) TGX gels (Bio-rad), and after the gels were stained with Bio-Safe^™^ Coomassie G-250 Stain (Bio-rad), they were dried with HydroTech^™^ Gel Drying System (Bio-rad). Radioactive proteins were detected using a Typhoon^™^ FLA-7000 system (GE Healthcare).

To identify phosphorylation sites on TDM1, kinase reactions were carried out with CDKA;1-TAM, 2 μg GST-TDM1-His6, 1 mM ATP (Sigma) per reaction in kinase buffer with a final volume of 20 μl. After incubation for 1 h at 30°C, the reactions were stopped by adding Laemmli sample buffer and boiled. Samples were separated on 7.5% TGX gels, and the gels were stained with Bio-Safe^™^ Coomassie G-250 Stain. An LTQ-Orbitrap XL (Thermo Fisher Scientific) coupled with an EASY-nLC 1000 (Thermo Fisher Scientific) was used for nano-LC-MS/MS analyses as described previously [45].

## Supporting Information

S1 Fig Alignment of the TDM1 and TDM1-like1 proteins. TDM1 and TDM1-like1 proteins from representative plant species (S2 Fig) were aligned with the T-Coffee algorithm. The residues identical or similar in at least 70% of the aligned proteins are shaded with a colour code. The conserved consensus sequence of the region comprising the T16P17 motif is boxed is shown (http://weblogo.berkeley.edu/logo.cgi). Three other putative CDK phosphorylation sites ([S/T]-P) are indicated by a star. The region containing four predicted TPR domains is indicated by a green line. Analyses were performed using the MPI toolkit with default parameters and formatted with Bioedit. *At*: *Arabidopsis thaliana*. *Al Arabidopsis lyrata*. *Bra*: *Brassica rapa*. *Sly*: *Solanum lycopersicum*. *St*: *Solanum tuberosum*. *Csa*: *Cucumis sativus*. *Eucgr*: *Eucalypsus grandis*. *Cp Carica papaya*. *ME*: *Manihot esculenta*. *TC*: *Theobroma cacao*. *Goraii*: *Gossypium raimondii*. *FV*: *Fragaria vesca*. *Pp*: *Prunus persica*. *LJ*: *Lotus japonicus*. *MT*: *medicago truncatula*. *GM*: *Glycine max*. *Pv*: *Phaseolus vulgaris*. *VV*: *Vitis vinifera Aq*: *Aquilegia coerulea*. *OS*: *Oriza sativa japonica*. *OSINDICA*: *Oriza sativa indica*. *BD*: *Brachypodium distachyon*. *SB*: *Sorghum bicolor*. *ZM*: *Zea mays*. *Si*: *Setaria italica*.
(PDF)

S2 Fig Phylogenetic tree of the plant TDM1 and TDM1 homologues. Proteins of the TDM1 clade are shown for all species. More distant TDM1 homologs are shown only for *A*. *thaliana* and *Brachypodium distachyon*. The analysis was performed on the Phylogeny.fr platform and comprised the following steps. Sequences were aligned with T-Coffee (v6.85) using the following pair-wise alignment methods: the 10 best local alignments (Lalign_pair), an accurate global alignment (slow_pair). After alignment, positions with gap were removed from the alignment. The phylogenetic tree was reconstructed using the maximum likelihood method implemented in the PhyML program (v3.0 aLRT). *At*: *Arabidopsis thaliana*. *Al Arabidopsis lyrata*. *Bra*: *Brassica rapa*. *Sly*: *Solanum lycopersicum*. *St*: *Solanum tuberosum*. *Csa*: *Cucumis sativus*. *Eucgr*: *Eucalypsus grandis*. *Cp Carica papaya*. *ME*: *Manihot esculenta*. *TC*: *Theobroma cacao*. *Goraii*: *Gossypium raimondii*. *FV*: *Fragaria vesca*. *Pp*: *Prunus persica*. *LJ*: *Lotus japonicus*. *MT*: *medicago truncatula*. *GM*: *Glycine max*. *Pv*: *Phaseolus vulgaris*. *VV*: *Vitis vinifera Aq*: *Aquilegia coerulea*. *OS*: *Oriza sativa japonica*. *OSINDICA*: *Oriza sativa indica*. *BD*: *Brachypodium distachyon*. *SB*: *Sorghum bicolor*. *ZM*: *Zea mays*. *Si*: *Setaria italica*.
(PDF)

S3 Fig Mutating Pro17 abolishes the phosphorylation of Thr16 of TDM1 by CDKA;1-CYCA1;2/TAM complex *in vitro*. (A) Gel image of GST-TDM1-His6 variants analyzed by mass spectrometry. The wild type and the P17L mutant of TDM1 were subjected to CDKA;1-TAM kinase assays, without (-) and with (+) CDKA;1-TAM complex. Proteins were separated by SDS-PAGE after kinase reaction and stained with coomassie brilliant blue. Asterisks indicate protein contamination during the purification procedure. The color of the sample numbers correspond to the line colors of chromatograms in B to E. (B) Mass chromatograms (left) and mass spectrum (right) of the indicated peptide. The phosphopeptide 9-APPGVYYpTPPPAR-21 was only detected in the sample of TDM1-WT treated with CDKA;1-TAM (red) and not in the other samples. See also Fig 4. The red-colored “pT” indicates the phosphorylated threonine in the peptide sequence. (C) Mass chromatograms (left) and mass spectrum (right) of the indicated peptide. Corresponding to B, the non-phosphorylated peptide 9-APPGVYYTPPPAR-21 was only detected in the samples of TDM1-WT (black: without kinase activity added, and red: with kinase activity added); confirming the P-to-L point mutation, this peptide was not identified in the TDM1-P17L sample. See also Fig 4. (D) Mass chromatograms (left) and mass spectrum (right) of the indicated peptides. Corresponding to C, the peptide 9-APPGVYYTLPPAR-21 was only detected in the samples of TDM1-P17L (blue: without kinase activity added, and green: with kinase activity added), but not in the samples of TDM1-WT (black: without kinase activity added, and red: with kinase activity added). Importantly, the corresponding phosphorylated peptide, 9-APPGVYYpTLPPAR-21, was not observed in any samples. (E) Mass chromatograms (left) and mass spectrum (right) of the indicated peptides. The phosphopeptide 57-VPSGDpSPYVR-66 was detected in the samples of TDM1-WT and TDM1-P17L treated with CDKA;1-TAM kinase complexes (red and green, respectively), but not in the samples without the kinase treatment. The red-colored “pS”, indicates the phosphorylated serine in the peptide sequence. Mass chromatogram (B to E, left) is given by plotting the x-axis as the retention time and the y-axis as the ion peak intensity. Mass spectrum (B to E, right) is given by plotting the x-axis as the mass-to-charge ratio (m/z) and the y-axis as the ion peak intensity.
(TIF)

S4 Fig *OSD1* mutated in its seven S/T-P sites complements *osd1-3*. (A) OSD1 protein sequence. The seven amino-acids predicted to be potential CDK phosphorylation sites (S/T-P) appear in red. (B) Male meiotic products of *osd1-3* Dyads of spores are observed. (C) Male meiotic products of *osd1-3* transformed by a genomic clone of *OSD1* carrying seven mutations, changing the seven S/T-P sites into A-P. Tetrads were observed (5 independent transformants) showing that *OSD1* mutated in its seven S/T-P sites is still functional. Scale bar = 5μm.
(PDF)

S5 Fig The TDM1::Myc signal is specific. Immunolocalization of Myc (green) in (A) wild type and (B) in plant expressing TDM1::Myc. Pictures were taken and treated identically, except that the exposure time for the Myc signal was 1000 ms in wild type and 250ms in TDM1::Myc. In wild type the background signal is similar in meiocytes (m) and somatic cells (s). In TDM1::Myc plants, a strong signal is detected in meiocytes while no signal is detected in somatic cells. Scale bar = 10μm.
(TIF)
