# Supplementary material for: TDM1 Regulation Determines the Number of Meiotic Divisions
Source: PLoS Genet. 2016 Feb 12;12(2):e1005856. doi: 10.1371/journal.pgen.1005856 (PMC4752240; doi:10.1371/journal.pgen.1005856)
Supplement: S1 Fig — TDM1 and TDM1-like1 proteins from representative plant species (S2 Fig) were aligned with the T-Coffee algorithm. The residues identical or similar in at least 70% of the aligned proteins are shaded with a colour code. The conserved consensus sequence of the region comprising the T16P17 motif is boxed is shown (http://weblogo.berkeley.edu/logo.cgi). Three other putative CDK phosphorylation sites ([S/T]-P) are indicated by a star. The region containing four predicted TPR domains is indicated by a green line. Analyses were performed using the MPI toolkit with default parameters and formatted with Bioedit. At: Arabidopsis thaliana. Al Arabidopsis lyrata. Bra: Brassica rapa. Sly: Solanum lycopersicum. St: Solanum tuberosum. Csa: Cucumis sativus. Eucgr: Eucalypsus grandis. Cp Carica papaya. ME: Manihot esculenta. TC: Theobroma cacao. Goraii: Gossypium raimondii. FV: Fragaria vesca. Pp: Prunus persica. LJ: Lotus japonicus. MT: medicago truncatula. GM: Glycine max. Pv: Phaseolus vulgaris. VV: Vitis vinifera Aq: Aquilegia coerulea. OS: Oriza sativa japonica. OSINDICA: Oriza sativa indica. BD: Brachypodium distachyon. SB: Sorghum bicolor. ZM: Zea mays. Si: Setaria italica. (PDF) [file pgen.1005856.s001.pdf]

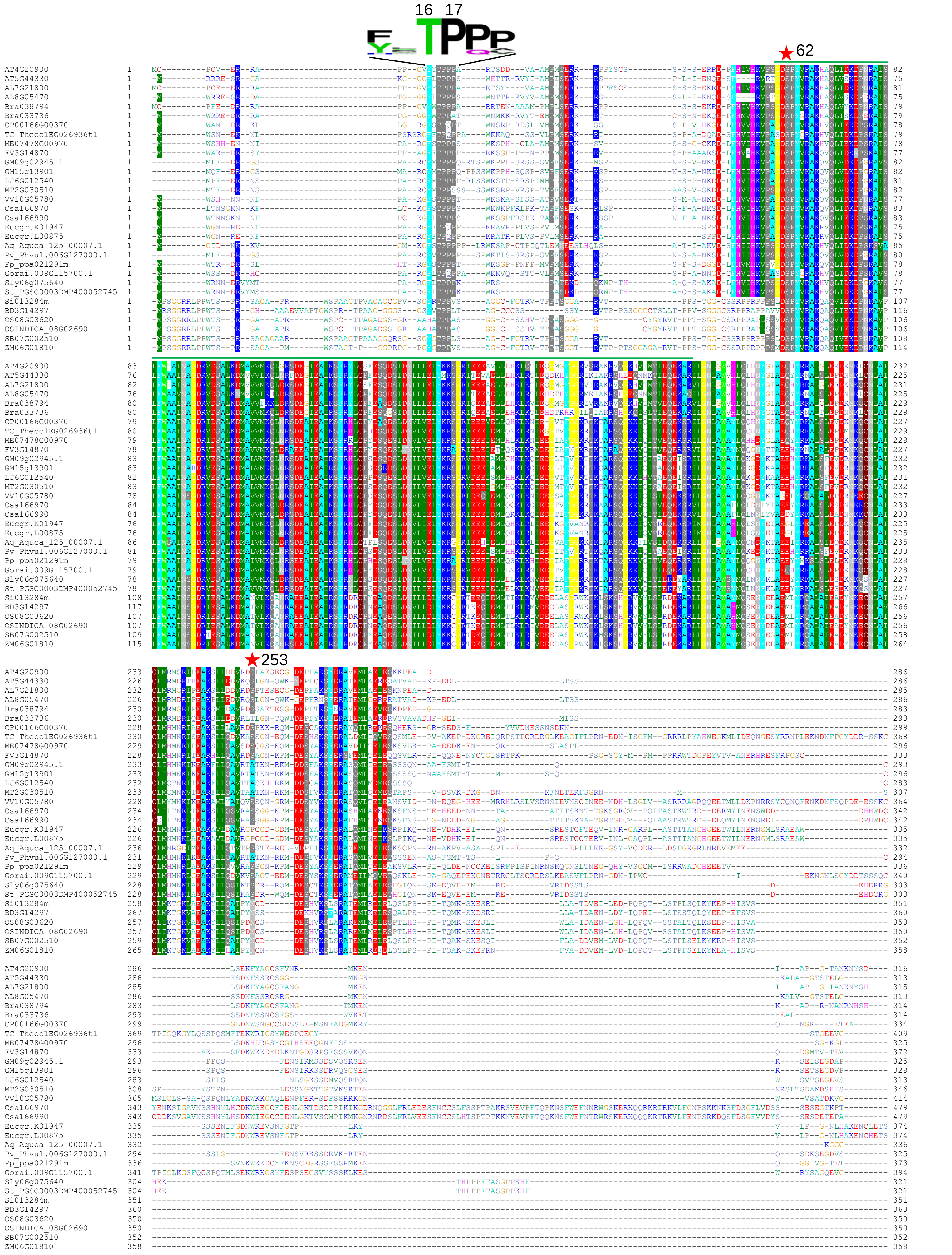
16 17
62
253
AT4G20900
AT5G44330
AL7G21800
AL8G05470
Bra038794
Bra033736
CP00166G00370
TC_Thecc1EG026936t1
ME07478G00970
FV3G14870
GM09g02945.1
GM15g13901
LJ6G012540
MT2G030510
VV10G05780
Csa166970
Csa166990
Eucgr.K01947
Eucgr.L00875
Aq_Aquca_125_00007.1
Pv_Phvul.006G127000.1
Pp_ppa021291m
Gorai.009G115700.1
Sly06g075640
St_PGSC0003DMP400052745
Si013284m
BD3G14297
OS08G03620
OSINDICA_08G02690
SB07G002510
ZM06G01810

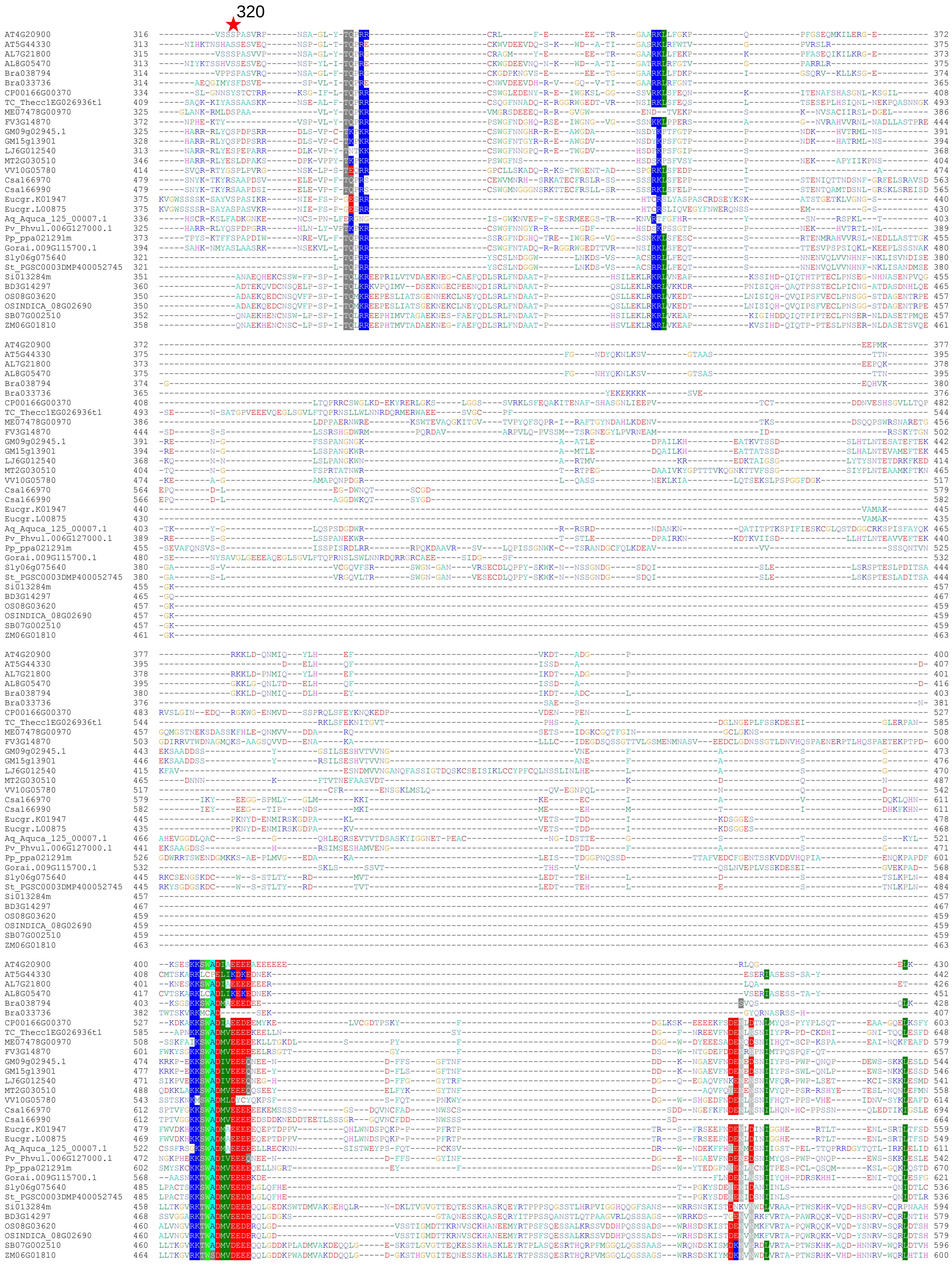
320
AT4G20900
AT5G44330
AL7G21800
AL8G05470
Bra038794
Bra033736
CP00166G00370
TC_Thecc1EG026936t1
ME07478G00970
FV3G14870
GM09g02945.1
GM15g13901
LJ6G012540
MT2G030510
VV10G05780
Csa166970
Csa166990
Eucgr.K01947
Eucgr.L00875
Aq_Aquca_125_00007.1
Pv_Phvul.006G127000.1
Pp_ppa021291m
Gorai.009G115700.1
Sly06g075640
St_PGSC0003DMP400052745
Si013284m
BD3G14297
OS08G03620
OSINDICA_08G02690
SB07G002510
ZM06G01810

```
AT4G20900                 430 ------------------------TAEM-------------------------------------------------------------------------------------------------------------------------- 434
AT5G44330                 442 ------------------------------------------------------------------------------AKI-----------TDIGQRKVLHIDQRKVRHIGQRS----------------------------------- 468
AL7G21800                 426 ------------------------------------------------------------------------------------------------------------------------------------------------------ 426
AL8G05470                 451 ------------------------------------------------------------------------------AKT-----------MDIGERKVL--------HIGRRS----------------------------------- 469
Bra038794                 428 ------------------------TAKI-------------------------------------------------------------------------------------------------------------------------- 432
Bra033736                 407 ------------------------------------------------------------------------------------------------------------------------------------------------------ 407
CP00166G00370             604 QKDGYNGTSG-NAVSS-R---N-PTARRSLYFGGDSNSLPLKEKD------------------------------SFT-GKN-----------ISMTRRNRLKVFTEITLLPDS------------------------------------- 669
TC_Thecc1EG026936t1       649 MKGGDNA-SA-NTVS--------------------------------------------------------------------------------SRRNRLPVFRDITSS---------------------------------------- 676
ME07478G00970             580 LKDGFAA-FS-NAVSP-R---N-PTVRRSLRF----DAK----------------------------------------ND-----------FSTRKKHRLQVFQDITPSIDS------------------------------------- 630
FV3G14870                 657 ---------------------------------------------------------------------------------------------QMKRRNRLQSFQDITN----------------------------------------- 673
GM09g02945.1              545 KKDGHYA-SE-SAILS-R---N-PTARRSLCF----NPELI--------------------------------------GE-----------KRLTRRSRLQVFQDITLLPETPRF---------------------------------- 600
GM15g13901                547 KKDDY-A-SE-STILS-R---N-PTARRSLCF----NPELI--------------------------------------GE-----------KKLTRRSRLQVFQDITLLPETPRF---------------------------------- 601
LJ6G012540                542 LKDGHHG-SG-TVTLS-R---K-PAVRRSLCF----NPELAKE-----------------------------RDSSL-SEE-----------KKPPRNNRLQVFKDIALHPETP------------------------------------ 603
MT2G030510                559 LKDGYNA-APVNDTWL-R---N-PTVRRSLFT----NAEMTNE-----------------------------RDVFS--GE-----------EKRTRRARLQVFQDITSSS--------------------------------------- 617
VV10G05780                615 LKDGYNT-PG-SDVSS-R---NNPTARRSLSN----ETM-ST----------------------------------S-GST-----------IRPKRRNRLQVFRDITLHTESPRT---------------------------------- 673
Csa166970                 695 IKDDS----D-EVVSS-R---N-SVERCPLYF----DQQQQPTLESIDNCCASPL-----PRKDL-T---TEVSCKF-GQE-----------NKLMRGNRLQVFHEITSVHQES------------------------------------ 773
Csa166990                 664 -------------------------------------------------------------------------------NV-----------EYKFNDETLTVHQELEC----------------------------------------- 683
Eucgr.K01947              560 LGCGSKS-QT-MDTPS-RTSGR-SAVRRSLNF----NQIPKQELDSV--MLLEPE-----KAGGCG---APEDAVPV-KKA-----------GQPPRRKRLQVFQDITRLPPHT------------------------------------ 643
Eucgr.L00875              550 LGCGSKS-QT-MDTPS-RTSGR-SAVRRSLNF----NQIPKQELDSV--MLLEPE-----KAGGCG---APEDAVPV-KKA-----------GQPPRRKRLQVFQDITRLPPHT------------------------------------ 633
Aq_Aquca_125_00007.1      612 LKKEEHI-KT-TASYSFS---N-PTARRTLAF----EKTDSAD-----YFRPSPLCKQVLF---------------N-GESDNTAGHGKNSMPVGRRSRRLPVFQEITP----------------------------------------- 689
Pv_Phvul.006G127000.1     543 QKDGYHP-SG-SVILS-R---N-PTARRSLCF----NPELSSESA--YAIRTSKSPKKASN---------LENRDTL-VGE-----------KKLPRKSRLQVFQDITLFPETPRF---------------------------------- 624
Pp_ppa021291m             671 LVDEY-V-SG-NAASS-R---N-STVRRSLCF----GQQQEQESVDY--ISSSPVPKEALNFEGSDSVQANGKGSIY-GKN-----------SSFSRRKRLQSFQDITEFQDS------------------------------------- 757
Gorai.009G115700.1        622 VKGGYNA-SA-NTVS--------------------------------------------------------------------------------LRRNRLQVFRDITST---------------------------------------- 649
Sly06g075640              537 LNEGY--------HTQ-P---G-REARRSLCF----DHNDRKEKCSS--GFQGKEL----KSGSLNSLP-PIGDIAY-QTP-----------VTLMRRNRLQVFRDITPESPKP------------------------------------ 614
St_PGSC0003DMP400052745   537 LNEGY--------HTQ-P---G-REARRSLCF----DQNDRKEKCSS--DFQGKVL----KSGSLNSLP-PIGDIAY-QTP-----------VTLMRRNRLQVFRDITPESPKP------------------------------------ 614
Si013284m                 595 LKENTSG---SKQAP-WR---S-SASQRALFP----DWKSKGEGYGH--GYVPFGDNEHSQGSSRTEA-THRWHNNAA-GT-----------VSWRPQNRLRVFQEITNEINQNVV---------------------------------- 683
BD3G14297                 580 LGEKAQGQ-GAEQTP-WR---S-SAAQRSLF---------------H--GHLPSGESGRCHVSGHTEA-VNRWPKNAAAIT-----------RPWRAQNRLRVFQEITNEMNRNVAYEHCCLPCMQTLCACTFWRYCLDVTAWFI--CCN 692
OS08G03620                580 LSDRAEG---TEQPP-WR---S-STAQRSLFP----DWKSKCERYGH--GYVPFGDNEHFQGSSHFEA-TNRWPKNAR---------------PWRPQNRLWVFQEITNEINQKQT-RALWLTIYTTL---LFGTTALDDTAWFLVECFH 695
OSINDICA_08G02690         580 LSDRAEG---TEQPP-WR---S-STAQRSLFP----DWKSKCERYGH--GYVPFCDNEHFQGSSHFEA-TNRWPKNAR---------------PWRPQNRLWVFQEITNEINQKQT-RALWLTIYTTL---LFGTTALDDTAWFLVECFH 695
SB07G002510               597 PHERASD---TNQVP-RR---S-NTSQRALFP----DWKSKGEGHGH--GCVLFDDNERTQCSSHVEA-THRWHNNEA-ST-----------GSWRPQNRLRVFQEITNEINQNVV---------------------------------- 685
ZM06G01810                601 PRERASG---TKQVP-RR---S-NTSQRALFP----DWKSKGEGYGH--GYVPFDDNEHTQCSSHIEAATHRWHNNEA-ST-----------GSWRPQNRLRVFQEITNEINQNVV---------------------------------- 690

AT4G20900                 434 ------------------------------------------------------------------------------------------------------------------------ 434
AT5G44330                 468 -----------------------------------------------------------------------------------------------------------------------V 469
AL7G21800                 426 ------------------------------------------------------------------------------------------------------------------------ 426
AL8G05470                 469 -----------------------------------------------------------------------------------------------------------------------V 470
Bra038794                 432 ------------------------------------------------------------------------------------------------------------------------ 432
Bra033736                 407 ------------------------------------------------------------------------------------------------------------------------ 407
CP00166G00370             669 -----------------------------------------------------------------------------------------------------------------------P 670
TC_Thecc1EG026936t1       676 ------------------------------------------------------------------------------------------------------------------------ 676
ME07478G00970             630 -----------------------------------------------------------------------------------------------------------------------P 631
FV3G14870                 673 ------------------------------------------------------------------------------------------------------------------------ 673
GM09g02945.1              600 -----------------------------------------------------------------------------------------------------------------------A 601
GM15g13901                601 -----------------------------------------------------------------------------------------------------------------------A 602
LJ6G012540                603 ------------------------------------------------------------------------------------------------------------------------ 603
MT2G030510                617 ------------------------------------------------------------------------------------------------------------------------ 617
VV10G05780                673 ------------------------------------------------------------------------------------------------------------------------ 673
Csa166970                 773 ------------------------------------------------------------------------------------------------------------------------ 773
Csa166990                 683 ------------------------------------------------------------------------------------------------------------------------ 683
Eucgr.K01947              643 -----------------------------------------------------------------------------------------------------------------------A 644
Eucgr.L00875              633 -----------------------------------------------------------------------------------------------------------------------T 634
Aq_Aquca_125_00007.1      689 ------------------------------------------------------------------------------------------------------------------------ 689
Pv_Phvul.006G127000.1     624 -----------------------------------------------------------------------------------------------------------------------A 625
Pp_ppa021291m             757 -----------------------------------------------------------------------------------------------------------------------P 758
Gorai.009G115700.1        649 ------------------------------------------------------------------------------------------------------------------------ 649
Sly06g075640              614 ------------------------------------------------------------------------------------------------------------------------ 614
St_PGSC0003DMP400052745   614 ------------------------------------------------------------------------------------------------------------------------ 614
Si013284m                 683 ------------------------------------------------------------------------------------------------------------------------ 683
BD3G14297                 693 VL--------------------------------------------------------------------HVAFICYCCCCWLAGWLAG------------QFPA--------------- 717
OS08G03620                696 LLAGHLLLHDGLQDLSVAPPSGQFAEMAMQMHPATGLSTHWSQVDSPPLKLREGANMVVLLQDQLLPMAFSIANVCEDCIMAYMSASASSVLFYTSCLDMQQLPYTGHVITINKSEAATI 815
OSINDICA_08G02690         696 LLAGHLLLHDGLQDLSVAPPSGQFAEMAMQMHPATGLSTHWSQVDSPPLKLREGANMVVLLQDQMLPMAFSIANVCEDCIMAYMSASASSVLFYTSCLDMQQLPYTGHVITINKSETA-- 813
SB07G002510               685 ------------------------------------------------------------------------------------------------------------------------ 685
ZM06G01810                690 ------------------------------------------------------------------------------------------------------------------------ 690
```
