# Supplementary figures and images for: TDM1 Regulation Determines the Number of Meiotic Divisions
Source: PLoS Genet. 2016 Feb 12;12(2):e1005856. doi: 10.1371/journal.pgen.1005856 (PMC4752240; doi:10.1371/journal.pgen.1005856)

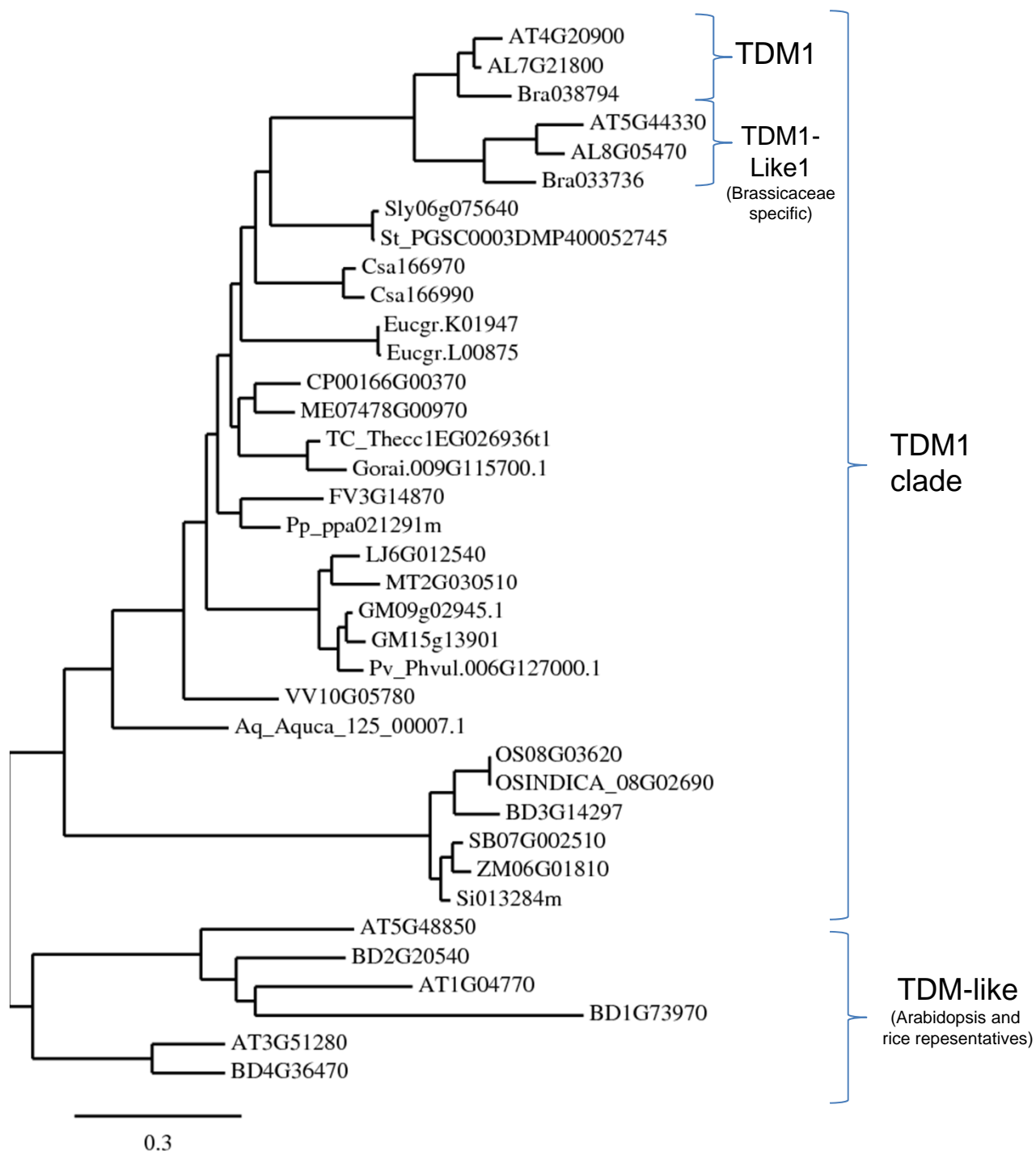

Supplement: S2 Fig — Proteins of the TDM1 clade are shown for all species. More distant TDM1 homologs are shown only for A. thaliana and Brachypodium distachyon. The analysis was performed on the Phylogeny.fr platform and comprised the following steps. Sequences were aligned with T-Coffee (v6.85) using the following pair-wise alignment methods: the 10 best local alignments (Lalign_pair), an accurate global alignment (slow_pair). After alignment, positions with gap were removed from the alignment. The phylogenetic tree was reconstructed using the maximum likelihood method implemented in the PhyML program (v3.0 aLRT). At: Arabidopsis thaliana. Al Arabidopsis lyrata. Bra: Brassica rapa. Sly: Solanum lycopersicum. St: Solanum tuberosum. Csa: Cucumis sativus. Eucgr: Eucalypsus grandis. Cp Carica papaya. ME: Manihot esculenta. TC: Theobroma cacao. Goraii: Gossypium raimondii. FV: Fragaria vesca. Pp: Prunus persica. LJ: Lotus japonicus. MT: medicago truncatula. GM: Glycine max. Pv: Phaseolus vulgaris. VV: Vitis vinifera Aq: Aquilegia coerulea. OS: Oriza sativa japonica. OSINDICA: Oriza sativa indica. BD: Brachypodium distachyon. SB: Sorghum bicolor. ZM: Zea mays. Si: Setaria italica. (PDF) [file pgen.1005856.s002.pdf]

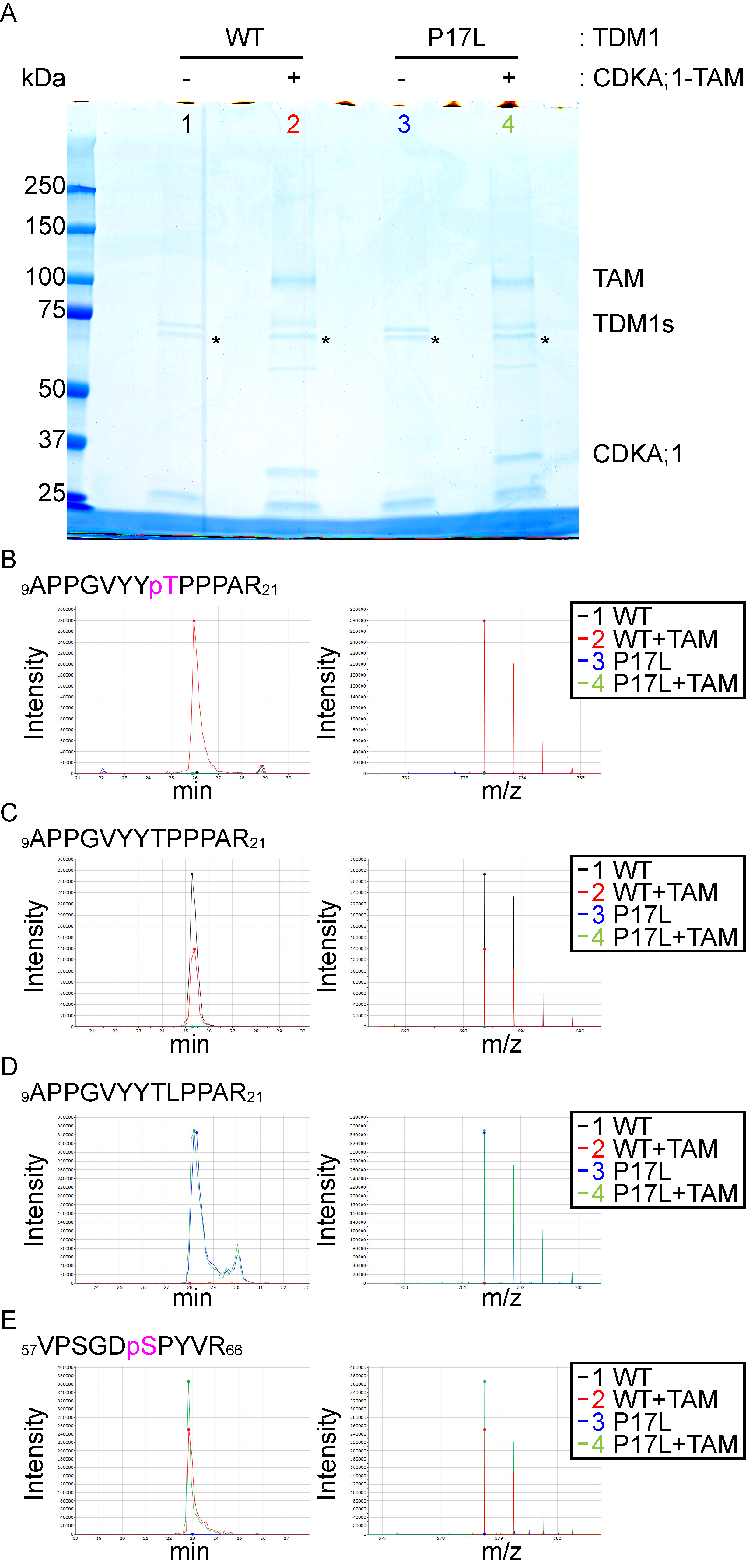

Supplement: S3 Fig — (A) Gel image of GST-TDM1-His6 variants analyzed by mass spectrometry. The wild type and the P17L mutant of TDM1 were subjected to CDKA;1-TAM kinase assays, without (-) and with (+) CDKA;1-TAM complex. Proteins were separated by SDS-PAGE after kinase reaction and stained with coomassie brilliant blue. Asterisks indicate protein contamination during the purification procedure. The color of the sample numbers correspond to the line colors of chromatograms in B to E. (B) Mass chromatograms (left) and mass spectrum (right) of the indicated peptide. The phosphopeptide 9-APPGVYYpTPPPAR-21 was only detected in the sample of TDM1-WT treated with CDKA;1-TAM (red) and not in the other samples. See also Fig 4. The red-colored “pT” indicates the phosphorylated threonine in the peptide sequence. (C) Mass chromatograms (left) and mass spectrum (right) of the indicated peptide. Corresponding to B, the non-phosphorylated peptide 9-APPGVYYTPPPAR-21 was only detected in the samples of TDM1-WT (black: without kinase activity added, and red: with kinase activity added); confirming the P-to-L point mutation, this peptide was not identified in the TDM1-P17L sample. See also Fig 4. (D) Mass chromatograms (left) and mass spectrum (right) of the indicated peptides. Corresponding to C, the peptide 9-APPGVYYTLPPAR-21 was only detected in the samples of TDM1-P17L (blue: without kinase activity added, and green: with kinase activity added), but not in the samples of TDM1-WT (black: without kinase activity added, and red: with kinase activity added). Importantly, the corresponding phosphorylated peptide, 9-APPGVYYpTLPPAR-21, was not observed in any samples. (E) Mass chromatograms (left) and mass spectrum (right) of the indicated peptides. The phosphopeptide 57-VPSGDpSPYVR-66 was detected in the samples of TDM1-WT and TDM1-P17L treated with CDKA;1-TAM kinase complexes (red and green, respectively), but not in the samples without the kinase treatment. The red-colored “pS”, indicates the phosphoryl [file pgen.1005856.s003.tif]

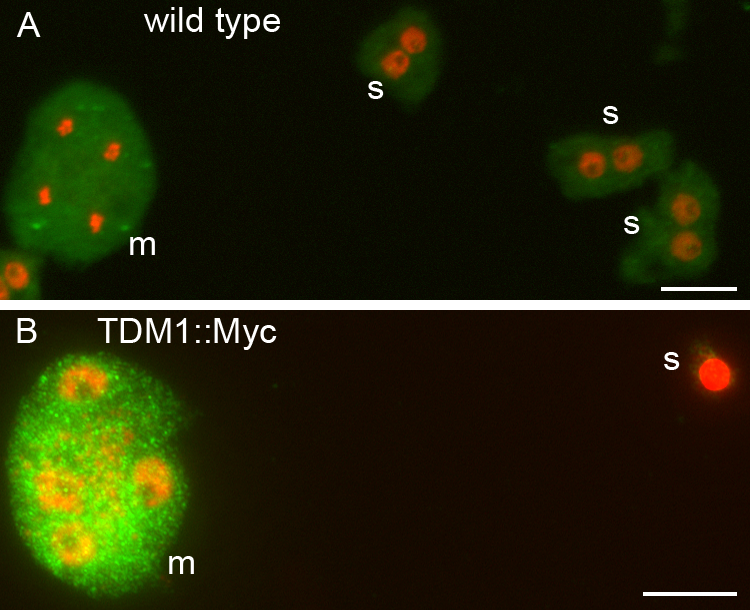

Supplement: S5 Fig — Immunolocalization of Myc (green) in (A) wild type and (B) in plant expressing TDM1::Myc. Pictures were taken and treated identically, except that the exposure time for the Myc signal was 1000 ms in wild type and 250ms in TDM1::Myc. In wild type the background signal is similar in meiocytes (m) and somatic cells (s). In TDM1::Myc plants, a strong signal is detected in meiocytes while no signal is detected in somatic cells. Scale bar = 10μm. (TIF) [file pgen.1005856.s005.tif]
