# Supplementary material for: TDM1 Regulation Determines the Number of Meiotic Divisions
Source: PLoS Genet. 2016 Feb 12;12(2):e1005856. doi: 10.1371/journal.pgen.1005856 (PMC4752240; doi:10.1371/journal.pgen.1005856)

**A**

MPEARDRTERPVDYSTIFANRRRHGILLDEPDS
RLSLIE**S**PVNPDIGSIGGTGGLVRGNFTTWRPG
NGRGGH**T**PFRLPQGRENMPIVTARRGRGGGLL
PSWYPR**T**PLRDITHIVRAIERRRGAGTGGDDGR
VIEIPTHRQVGVLE**S**PVPLSGEHKCSMV**T**PGPS
VGFKRSCPPSTAKVQKMLLDITKEIAEEEAGFI**T**P
EKKLLNSIDKVEKIVMAEIQKLKS**T**PQAKREERE
KRVRTLMTMR

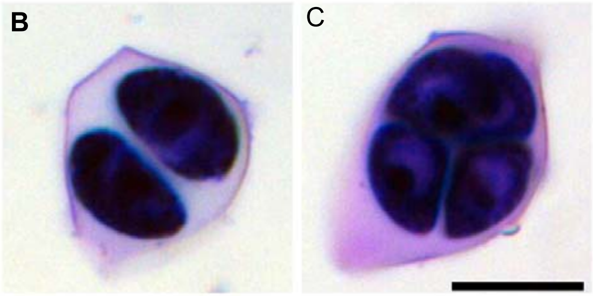

Supplement: S4 Fig — (A) OSD1 protein sequence. The seven amino-acids predicted to be potential CDK phosphorylation sites (S/T-P) appear in red. (B) Male meiotic products of osd1-3 Dyads of spores are observed. (C) Male meiotic products of osd1-3 transformed by a genomic clone of OSD1 carrying seven mutations, changing the seven S/T-P sites into A-P. Tetrads were observed (5 independent transformants) showing that OSD1 mutated in its seven S/T-P sites is still functional. Scale bar = 5μm. (PDF) [file pgen.1005856.s004.pdf]
